# Genomic and secretomic analyses of *Blastobotrys* yeasts reveal key xylanases for biomass decomposition

**DOI:** 10.1007/s00253-025-13556-5

**Published:** 2025-08-01

**Authors:** Jonas Ravn, Amanda S. Ristinmaa, Scott Mazurkewich, Guilherme B. Dias, Johan Larsbrink, Cecilia Geijer

**Affiliations:** 1 Department of Life Sciences, Division of Industrial Biotechnology, Chalmers University of Technology, 412 96 Gothenburg, Sweden; 2 Wallenberg Wood Science Center, Teknikringen 56-58, 100 44, Stockholm, Sweden; 3 Division of Bioeconomy, Sweden, Department of Food Research and Innovation, RISE Research Institutes of Sweden, Frans Perssons Väg 6, SE-412 76 Gothenburg, Sweden; 4 Department of Cell and Molecular Biology, National Bioinformatics Infrastructure Sweden, Science for Life Laboratory, Uppsala University, Husargatan 3, 75237 Uppsala, Sweden

**Keywords:** Xylanolytic yeast, Xylan, GH30, GH11, Glycoside hydrolase, Wood

## Abstract

**Abstract:**

Xylanolytic enzyme systems in ascomycetous yeasts remain underexplored, despite the presence of yeasts in various xylan-rich ecological niches. In this study, we investigated the secreted xylanolytic machineries of three *Blastobotrys* species—*B. mokoenaii*, *B. illinoisensis*, and *B. malaysiensis*—by integrating genome annotation, bioinformatics, and secretome analyses of cultures grown on beechwood glucuronoxylan. Our findings demonstrate that these yeasts effectively hydrolyze xylan through the secretion of xylanases from the glycoside hydrolase (GH) family 11, which play a central role in cleaving the xylan backbone. Additionally, the yeasts produce a diverse array of other CAZymes, including members of GH families 3, 5, and 67, with putative roles in xylan degradation. We also report on the heterologous expression and functional characterization of the GH30_7 xylanase *Bm*Xyn30A from *B. mokoenaii*, which exhibits both glucuronoxylanase and xylobiohydrolase activities. We demonstrate additive effects between GH family 30 *Bm*Xyn30A and GH family 11 *Bm*Xyn11A during the hydrolysis of beechwood glucuronoxylan, where the enzymes exhibit complementary roles that enhance the deconstruction of this complex hemicellulose substrate. These findings broaden our understanding of the xylanolytic systems in yeasts and underscore the potential of *Blastobotrys* species as cell factories and natural xylanase producers. The enzymes they produce hold promise for biorefining applications, enabling efficient utilization of renewable xylan-rich plant biomass resources.

**Key points:**

•* Extracellular GH11 xylanases dominate glucuronoxylan degradation in Blastobotrys yeasts.*

•* Yeast GH30_7 enzyme shows multifaceted activity, supporting complex xylan breakdown.*

•* Blastobotrys yeasts show promise as cell factories for industrial biotechnology applications.*

**Supplementary Information:**

The online version contains supplementary material available at 10.1007/s00253-025-13556-5.

## Introduction

The diversity of yeast in nature is immense, with an estimated 20,000–200,000 species with varied nutritional requirements and abilities to metabolize a wide array of carbon substrates (Boekhout et al. 2022). While over 100 yeast species with xylan-degrading capabilities have been identified (Šuchová et al. 2022b), their enzymatic mechanisms and ecological roles remain underexplored compared to bacteria and filamentous fungi. These yeasts may have significant cell factory potential, as they can combine enzymatic xylan degradation with the conversion of released xylose sugars into valuable bioproducts. This could reduce the need for harsh biomass pretreatments and lower the energy demands in lignocellulosic biomass processing in industrial biotechnology applications (Banner et al. 2021). However, a deeper understanding of the specific xylan degradation mechanisms employed by these yeasts is fundamental to grasping their role in carbon cycling.

Xylans are key components of plant cell walls, consisting of a β−1,4-linked d-xylose backbone that is often extensively *O*-acylated and substituted with groups such as α−1,2-linked (methyl)-glucuronic acid, α−1,2- or α−1,3-linked l-arabinosyl units, and phenolic compounds (Sjöström 1993; Mnich et al. 2020). This structural diversity gives rise to various xylan types, including glucuronoxylan (GX), which coats cellulose fibrils and acts as a physical barrier inhibiting cellulases (Simmons et al. 2016; Curry et al. 2023; Zexer et al. 2024). GX-rich agricultural and forestry side streams offer abundant feedstocks for microbial conversion into value-added bioproducts (Naidu et al. 2018).

Xylan-degrading microorganisms, including yeasts, use Carbohydrate-Active EnZymes (CAZymes), collected and described within the CAZy database (www.cazy.org; Drula et al. 2022), to degrade xylan polymers into monosaccharides that can be further catabolized and used as carbon and energy sources. Xylanases are the key enzymes responsible for the deconstruction of xylan by cleaving the β−1,4-linked polysaccharide backbone by endo-acting mechanisms. The primary xylanase enzyme families, classified as glycoside hydrolases (GHs), include GH families 5, 10, 11, and 30 (Collins et al. 2005; Mendonça et al. 2023). Overall, xylanase activity can be increased by the activity of other debranching enzymes that improve substrate access by stripping away side chain substitutions, including α-glucuronidases (e.g., GH67 and GH115), α-l-arabinofuranosidases (e.g., GH43, GH51, GH62), and carbohydrate esterases (CEs) (e.g., CE1, CE4, CE9) (Jia et al. 2016; Puchart and Biely 2022). Finally, xylanase-generated xylooligosaccharides are hydrolyzed by β-xylosidases (e.g., GH1, GH3, GH5, GH43, GH51) into xylose monomers that can be taken up and metabolized within the cell (Rohman et al. 2019; Kojima et al. 2022).

A previous study mapped CAZymes in 332 genome-sequenced ascomycetous yeasts, identifying the *Trichomonascaceae* clade as a phylogenetic hotspot for CAZyme-rich species (Ravn et al. 2021). Several yeasts in this clade also exhibit biotechnologically relevant traits, particularly *Blastobotrys adeninivorans* and the closely related *Blastobotrys raffinosifermentans*, known for efficient protein secretion (Du Preez et al. 2009) and lipid accumulation (Ruben et al. 2021). Additionally, *Blastobotrys mokoenaii* and *Sugiyamaella lignohabitans* excel in xylan degradation. Bioinformatic analysis predicts that these yeasts possess several xylan-degrading enzymes, and two of their respective xylanases, *Bm*Xyn11A and *Sl*Xyn30A, have been recombinantly expressed and characterized (Ravn et al. 2023; Šuchová et al. 2022a). However, the lack of proteome and transcriptome studies, as well as insufficient elucidation of genotype/phenotype associations, hampers our understanding regarding which of the predicted enzymes are actively involved in xylan deconstruction in these yeasts.

Phylogenetic association with *B. mokoenaii* led to the identification of two additional *Blastobotrys* species, *B. illinoisensis* and *B. malaysiensis*, which exhibited marked and robust xylanolytic capabilities comparable to those of *B. mokoenaii* (Ravn et al. 2021). However, no genomes for *B. illinoisensis* and *B. malaysiensis* were available at that time, which limited further analysis and exploration of their xylanolytic enzymes. Therefore, the aim of this study was to combine genomics, secretomics, and growth and enzymatic activity assays to investigate the xylanolytic capacities of the three *Blastobotrys* yeasts, shedding light into the enzymatic mechanisms underlying their efficient metabolism of GX.

## Methods

### Yeast strains

*B. mokoenaii* CBS8435 (Y-27120) isolated from savanna soil in South Africa, *B. illinoisensis* CBS 10339 (YB-1343) isolated from a tree in Illinois, USA, and *B. malaysiensis* CBS 10336 (Y-6417) isolated from cave soil in Malaysia, were ordered from the ARS Culture Collection, Peoria, IL, USA (NRRL; https://nrrl.ncaur.usda.gov/). Strains were revived according to instructions from the provider and stored in 30% glycerol at − 80 °C.

### Genome sequencing

*B. illinoisensis* and *B. malaysiensis* were grown in YPD overnight at 30 °C, 200 rpm, and cells were washed in milliQ purified water and harvested using centrifugation (6000 × g, 10 min). Cells were lysed using zymolyase-20T treatment at 37 °C, 30 min while buffered in 1 M sorbitol, 0.1 M ethylenediaminetetraacetic acid (EDTA)-Na^2^ at pH 7.5. DNA was extracted by adding 10 mL/g cell mix of 2% cetyltrimethylammonium bromide (CTAB) buffer (100 mM Tris–HCl, pH 8.0, 20 mM EDTA with 1.4 M NaCl), which was briefly vortexed and incubated at 57 °C for 1 h. DNA was extracted three times using phenol/chloroform and the 2-propanol precipitation method as described previously (Tõlgo et al. 2021). To remove residual RNA, genomic DNA was incubated with 200 µg mL^−1^ RNase A (Thermo Fisher Scientific, Waltham, MA, USA) at 60 °C for 2 h. Extracted genomic DNA was purified further using NucleoSpin soil kit (Macherey–Nagel, Düren, Germany), and DNA-RNA quality was analyzed using Qubit dsDNA HS Assay kit (Thermo Fisher Scientific, Waltham, MA, USA). Approximately ~ 10 µg of high molecular weight genomic DNA was sent for PacBio NGS sequencing at SciLifeLab in Uppsala, Sweden. Library prep was performed using the SMRTbell Template Prep Kit according to the manufacturer’s instructions and sequenced on a single SMRT cell on a PacBio Sequel instrument (PacBio, Menlo Park, CA, USA).

### Genome assembly and annotation

Whole-genome assembly was performed using the microbial assembly pipeline in SMRT Link v10 which uses HGAP4 for assembly and one round of polishing with arrow (Chin et al. 2013). Genome assembly statistics were computed using gfastats v1.3.6 (Formenti et al. 2022), and Benchmarking of Universal Single-Copy Orthologs (BUSCO) was performed using BUSCO v5.7.1 (Manni et al. 2021) with the saccharomycetes_odb10 gene set from OrthoDB v10 (Kriventseva et al. 2019). Prior to gene annotation, the polished assemblies were processed with RepeatModeler2 (Bourque et al. 2018) to generate a species-specific repeat library and then masked with RepeatMasker. Gene annotation was performed with Braker v3.0.3 (Gabriel et al. 2024), incorporating evidence in the form of all fungal proteins from OrthoDB v11 (Kuznetsov et al. 2023). The predicted genes were functionally annotated using the NBIS functional annotation nextflow pipeline v2.0.0 (https://github.com/NBISweden/pipelines-nextflow). Briefly, this pipeline performs similarity searches between the annotated proteins and the UniProtKB/Swiss-Prot database (Magrane and Consortium 2011) (downloaded on 2023–12) using BLAST. Then, it uses InterProScan (Jones et al. 2014) to query the proteins against InterPro (Paysan-Lafosse et al. 2023) v59-91 databases and merges results using AGAT v1.2.0 (10.5281/zenodo.8178877). Transfer RNA (tRNA) and ribosomal RNA (rRNA) genes were annotated using tRNAscan-SE (Chan et al. 2021) v2.0.12 and barrnap v0.9 (https://github.com/tseemann/barrnap), respectively.

### Comparative genomics

Pairwise average nucleotide identity (ANI) values were obtained from the whole-genome sequences of all species using fastANI (Jain et al. 2018) v1.34. OrthoFinder v2.5.5 was used to estimate the numbers of genes that could be clustered between species (Emms and Kelly 2019).

### CAZyme prediction

Predicted protein-coding sequences from BRAKER v3.0.3 were used to predict CAZymes using the dbCAN3 server (https://bcb.unl.edu/dbCAN2/) (Zheng et al. 2023).

### Yeast growth on different carbon sources

Yeasts were pre-cultured overnight at 30 °C, shaking 200 rpm in autoclaved liquid yeast extract–peptone–dextrose (YPD) containing 10 g L^−1^ yeast extract, 20 g L^−1^ peptone, and 20 g L^−1^ glucose. Growth on glucose, xylose, arabinose, galactose, lactose, and mannose (Sigma-Aldrich, Schnelldorf, Germany and Merck Rahway, NJ, USA); beechwood glucuronoxylan (Megazyme, Bray, Ireland); wheat arabinoxylan (Megazyme, Bray, Ireland); mixed-linkage β−1,3/1,4-glucan (barley, Megazyme, Bray, Ireland); glucomannan (konjac, Sigma-Aldrich, Schnelldorf, Germany); xyloglucan (tamarind, Megazyme, Bray, Ireland); and carboxymethyl cellulose (Sigma-Aldrich, Schnelldorf, Germany) was carried out in sterile Delft minimal media containing 5 g L^−1^ ammonium sulfate, 3 g L^−1^ potassium phosphate, 1 g L^−1^ magnesium sulfate, vitamins, and trace metals adjusted to pH 5 with 2 M KOH (Hendriks et al. 2018). After cell harvest and washing (4500 rpm, 5 min) of pre-cultures, yeasts were inoculated with a starting OD_600_ = 0.1 in liquid Delft media containing the different carbon sources at a concentration of 0.5–2% (w/v). Yeast growth in liquid cultures was monitored in triplicates over time at 30 °C and 200 rpm using a 96-well plate setup in a Growth-Profiler 960 (EnzyScreen, Heemstede, Netherlands). Agar plates containing 4 g L^−1^ (0.4%) carbon source and 20 g L^−1^ agar were inoculated with a 20 µL drop of cell suspension with a cell density of OD_600_ = 5 acquired from 3 × washed (8000 rpm, 10 min) 10 mL YPD yeast pre-cultures, spotted in the middle of the agar plate. Plates were kept at room temperature for 5 weeks while monitoring growth daily using an EPSON perfection V800 scanner (EPSON, Nagano, Japan) with a customized plate scaffold. The agar plate picture brightness was edited using the Affinity Photo 2 software (West Bridgford, UK).

### Secretome activity in xylan-grown cultures

Yeasts were assayed for secreted xylanolytic activity over time from triplicates of 30-mL Delft media containing 20 g L^−1^ beechwood GX enriched liquid cultures using 100 µL samples which were centrifuged (10,000 × g, 5 min) at 4 °C and immediately frozen to − 20 °C after sampling. Endo-1,4-β-xylanase activity was quantified using a 250 µL mixture of thawed 25 µL cell-free supernatant mixed with fresh 10 g L^−1^ beechwood GX (Megazyme, Bray, Ireland) suspension buffered in 50 mM sodium acetate (pH 5). The buffered mixture was incubated for 30 min at 30 °C and 600 rpm followed by immediate chilling on ice and inactivation at 98 °C for 5 min. Reducing sugar contents were determined using the dinitrosalicylic acid method (McCleary and McGeough 2015). Secreted β-xylosidase activity from cell-free beechwood GX culture supernatants was quantified from 250 µL reactions containing 25 µL cell-free supernatant and 5 mM *p*-nitrophenyl-β-d-xylopyranoside buffered in 50 mM sodium phosphate (pH 7) in a 96-well plate and incubated for 30 min at 30 °C and 600 rpm. Spectroscopic quantification of *p*-nitrophenol was performed at 405 nm.

### Secretomics and LC–MS/MS

Samples for secretome analysis were obtained from 30 mL Delft cultures with 20 g L^−1^ beechwood GX as sole carbon source, incubated at 30 °C, 200 rpm, and sampled after 72 h. Cell-free supernatants (12,000 × g, 5 min) were filtered through a 0.2 µm filter and frozen at − 20 °C until further processing. Samples were transferred to 15 mL 10 kDa Amicon spin columns (MilliPoreSigma, Burlington, MA, USA) and concentrated tenfold and buffer exchanged into sterile 50 mM sodium acetate buffer pH 5 with 50 mM NaCl. Protein concentration was measured using Nanodrop 2000 at 280 nm. Proteomics was performed by the Proteomics Core Facility, Sahlgrenska Academy, University of Gothenburg. Sample preparation was done using dl-dithiothreitol to reduce disulfide bonds and thereafter processed using the modified filter-aided sample preparation (FASP) method (Wisniewski et. al. 2009). Samples were analyzed by liquid chromatography coupled with tandem mass spectrometry (LC–MS/MS) using an Orbitrap Fusion Lumos Tribrid mass spectrometer with the FAIMS Pro ion mobility system, coupled with an Easy-nLC 1200 liquid chromatography system (Thermo Fisher Scientific, Waltham, MA, USA). Proteome Discoverer version 3.0 (Thermo Fisher Scientific, Waltham, MA, USA) was used for protein identification and relative quantification. The database search was performed using the Sequest search engine against custom databases with a precursor tolerance of 10 ppm and a fragment ion tolerance of 0.02 Da. Tryptic peptides were accepted with one missed cleavage; methionine oxidation was set as a variable modification, and cysteine carbamidomethylation was set as a fixed modification. Percolator was used for peptide spectrum matches (PSM) validation with a strict false discovery rate (FDR) threshold of 1%. Proteins were required to pass a protein FDR of 5%. LC–MS features were identified by the Minora Feature Detector node (Thermo Fisher Scientific, Waltham, MA, USA). Chromatographic alignment and feature mapping were enabled with a maximum RT shift of 5 min and a minimal signal-to-noise threshold of 5. Primary ion-intensity values at peak maximum for all unique peptides were used to calculate the corresponding protein abundances. Protein identification and analysis were processed using Pfam from the EGGNOG mapper available at www.galaxy.org, and signal peptides were predicted using the SignalP 6.0 online tool (https://services.healthtech.dtu.dk/services/SignalP-6.0/). Only proteins present in ≥ 2 of the three biological replicates, after filtering all search results to reach a protein FDR of 1%, were used for further analysis.

### Zymogram analysis

Zymograms were produced by soaking pre-cast SDS-PAGE gels (Bio-rad, Hercules, CA, USA) loaded with 5–10 µL tenfold concentrated secretomes from the *Blastobotrys* beechwood GX enriched cultures in 100 mL 2% w/v Triton X-100 followed by 30-min shaking at 100 rpm twice to remove SDS. The protein gel was incubated in 100 mL of 0.1 M sodium acetate buffer at pH 5 for 15 min twice before soaking in 100 mL of 20 g L^−1^ beechwood GX buffered in 0.1 M sodium acetate at pH 5 for 60 min at 50 °C. After soaking with xylan, the gel was washed in 200 mL deionized water five times at room temperature at 60 rpm. The gel was subsequently stained with 100 mL 0.1% Congo red for 30 min and finally washed twice with 200 mL of 1 M NaCl for 15 min before fixing in 3% acetic acid and stored in the dark. Gels were visualized using regular photography on a plate with a backlight.

### Heterologous protein expression

*Bm*Xyn30A (sequence ID in Supplementary List S1) was codon optimized and synthesized by GenScript (Rijswijk, Netherlands) after removal of the native signal peptide sequence for heterologous expression in *Pichia pastoris* X-33 and delivered in the pPICZa A vector (Invitrogen, Waltham, MA, USA). Recombinant pPICZa A constructs were coded for the yeast alpha-secretion factor, candidate gene, and a C-terminal His_6_ tag and were transformed into *Escherichia coli* DH5α One Shot Top10 cells (Invitrogen, Waltham, MA, USA). Transformants were selected using 25 mg mL^−1^ Zeocin in low-salt Luria–Bertani (LB) medium with 80 mM Tris–HCl at pH 7.5. The vector was propagated in 2 mL low-salt LB medium with 80 mM Tris–HCl, pH 7.5, with 25 mg mL^−1^ Zeocin and isolated using a GeneJET PCR purification kit (Thermo Fisher Scientific, Waltham, MA, USA). *P. pastoris* X-33 cells were transformed using 10 µg linearized (*Sac*I restriction enzyme) and 96% ethanol precipitated pPICZa A using electroporation, and clones were selected using 100 µg mL^−1^ Zeocin on yeast extract–peptone–dextrose (YPD) plates containing 1 M sorbitol. Genomic integration of recombinant vectors were confirmed by colony PCR using primers aligning to the alpha-factor and *AOX1* terminator plasmid parts. Positive *P. pastoris* X-33 clones were grown in medium scale at 20 mL, at 150 rpm and 25 °C in 100 mL baffled shake flasks in rich buffered glycerol-complex medium (BMGY). Methanol (1%, vol/vol) was used to induce expression in buffered methanol-complex medium (BMMY) over 4 days before harvesting as described in the EasySelect Pichia Expression kit (Thermo Fisher Scientific, Waltham, MA, USA).

### Enzyme purification

Recombinant *Bm*Xyn30A and *Bm*Xyn11A proteins were purified by immobilized metal affinity chromatography (IMAC) using 2 × 5 mL Ni-Sepharose excel resin (GE Healthcare, Chicago, IL, USA) in gravity columns. After sample loading, the column was washed with five column volumes of loading buffer (50 mM Tris, pH 8, 250 mM NaCl) before elution of His_6_-tagged proteins with loading buffer containing 250 mM imidazole. Protein purity was evaluated by SDS-PAGE (Supplementary Fig. S1), and a NanoDrop 2000 spectrophotometer (Thermo Fisher Scientific, Waltham, MA, USA) was used to determine protein concentration using the predicted molecular weights and extinction coefficients (Expasy ProtParam server, Swiss Institute of Bioinformatics).

### Biochemical characterization of BmXyn30A and xylan degradation assays

*Bm*Xyn30A was biochemically characterized using a 200 µL mixture of 10 g L^−1^ beechwood GX (Megazyme, Bray, Ireland) in 50 mM sodium acetate buffer (pH 5) using 10 µL non-diluted purified enzyme with a specific enzyme concentration of 1.5 mg mL^−1^. The mixture was incubated for 60 min, with measurements taken at 0, 10, 20, 30, and 60 min, followed by immediate chilling on ice and inactivation at 98 °C for 5 min, before reducing sugars were quantified using the dinitrosalicylic acid (DNS) method, as described earlier (McCleary and McGeough 2015). For pH optimum measurements, either a 100 mM sodium citrate buffer with a pH range of 3–6 or a 100 mM sodium phosphate buffer with a pH range of 6–8 was used, and activity was quantified by the DNS method. Assays for the determination of the enzyme’s optimum temperature were carried out in 100 mM sodium acetate at pH 5, and reducing sugars were quantified by the DNS method.

Degradation assays were used to determine enzymatic additive effects in xylan hydrolysis, combining *Bm*Xyn30A and *Bm*Xyn11A with β-xylosidase GH43 (Xyl43) from *Selenomonas ruminantium* (cat. no. E-BXSR; GH43, Megazyme, Bray, Ireland) and α-methyl-glucuronidase *Bo*Agu115A (Agu115) from *Bacteroides ovatus* (cat. no. CZO311; GH115, NZYTech, Lisbon, Portugal). A 1:1 molar ratio of *Bm*Xyn11A and *Bm*Xyn30A was used. Enzymes were incubated at normalized 0.1 µM concentrations in 400-µL mixtures of 10 g L^−1^ beechwood GX (Megazyme, Bray, Ireland) in 50 mM sodium acetate buffer, pH 5, for 16 h at 40 °C and 650 rpm, and then inactivated for 5 min at 98 °C before analysis by the DNS method.

### Xylooligosaccharide analysis by ion chromatography

The formation of xylooligosaccharides from enzymatic hydrolysis of 10 g L^−1^ beechwood GX (Megazyme, Bray, Ireland) by *Bm*Xyn30A and *Bm*Xyn11A combined with Agu115 and Xyl43 was analyzed after 16 h reactions (0.1 µM enzyme concentration in 10 g L^−1^ beechwood GX and 50 mM sodium acetate pH 5, at 40 °C and 650 rpm in 400 µL total volume). Samples were inactivated at 98 °C for 5 min before supernatants were filtered through a 0.2 µm filter and diluted twofold in sterile water and stored at 4 °C. Analysis was performed using a high-performance anion-exchange chromatography coupled with pulsed amperometric detection (HPAEC-PAD) and an ICS-5000 system (Dionex Sigma-Aldrich, Sunnyvale, CA, USA), operating at 25 °C. A CarboPac PA200 (250 mm by 3 mm) column (Thermo Fisher Scientific, Waltham, MA, USA) was used for separation of hydrolysis products with a gradient of eluents using a flow rate of 0.5 mL min^−1^: A, milliQ water; B, 300 mM sodium hydroxide; and C, 100 mM sodium hydroxide and 1 M sodium acetate. Standards (5 to 800 µM) of xylose (X1), xylobiose (X2), xylotriose (X3), xylotetraose (X4), xylopentaose (X5), and xylohexaose (X6) (Megazyme, Bray, Ireland) were used for quantitation. Xylobiohydrolase activity by *Bm*Xyn30A was analyzed using 200 µM xylotetraose in 50 mM sodium acetate buffer, pH 5, with time-course measurements taken at 0, 30, 60, 90, and 120 min, which were inactivated by 5 min incubation at 98 °C prior to HPAEC-PAD analysis.

## Results

### *Blastobotrys* genome sequencing and analysis

While the PacBio genome sequencing of *B. malaysiensis* was unsuccess for unknown reasons*, *a high-quality genome for *B. illinoisensis* was assembled, achieving 345-fold coverage and generating seven polished contigs. The genome shows high completeness with a BUSCO score of 94%, and the final assembly spans 14.3 Mbp with a G + C content of 50% (Table 1). For validation, the assembly was compared with the recently published *B. illinoisensis* genome assembled from short-read sequencing data, showing strong agreement in gene content and structure despite markedly different contiguity levels (7 contigs vs. 3550), as well as with assemblies for *B. mokoenaii* and *B. malaysiensis* (Table 1). These results provide a robust genomic foundation for subsequent comparative and functional analyses.

**Table 1:** Genome assembly statistics for the three *Blastobotrys* species

Stats	*B. mokoenaii* (Illumina)	*B. illinoisensis* (PacBio, this study)	*B. illinoisensis* (Illumina)	*B. malaysiensis* (Illumina)
Assembly length (bp)	13,688,864	14,346,273	16,309,197	15,805,505
# of contigs	333	7	3550	3421
Contig N50 (bp)	249,159	3,252,363	93,981	29,423
# of scaffolds	333	7	3538	2553
Scaffold N50 (bp)	249,159	3,252,363	111,420	118,479
GC content (%)	48.92	51.14	50.79	50.67
BUSCO (*Saccharomycetes*, *n* = 2137)	C: 94.4% (S: 93.7%, D: 0.7%), F: 2.5%, M: 3.1%	C: 94.3% (S: 93.8%, D: 0.5%), F: 2.8%, M: 2.9%	C: 94.0% (S: 92.0%, D: 2.0%), F: 2.9%, M: 3.1%	C: 92.4% (S: 91.0%, D: 1.4%), F: 4.0%, M: 3.6%
Reference	Shen et al. 2018	This study	Opulente et al. 2024	Opulente et al. 2024
NCBI accession number	GCA_003705765.3	GCA_965113335.1	GCA_030558835.1	GCA_030558815.1

The string of BUSCO results is abbreviated for brevity as follows: *C*, complete; *S*, complete and single-copy; *D*, complete and duplicated; *F*, fragmented; *M*, missing. *BUSCO*, Benchmarking of Universal Single-Copy Orthologs

Phylogenetic analysis of species in the *Trichomonascacea* clade placed the three *Blastobotrys* species close together (Visagie et al. 2023). On the Internal Transcribed Spacer (ITS) sequence level, *B. mokoenaii* shares 96.06% and 98.54% sequence identity with *B. illinoisensis* and *B. malaysiensis*, respectively, while *B. malaysiensis* and *B. illinoisensis* share 97.40% ITS sequence identity. Some of these percentages are close to the 98.41% identity threshold suggested by Vu et al. (2016) to delineate yeast species. To determine the genetic distance between the strains on a genomic level, the average nucleotide identities (ANI) were calculated using fastANI v1.34 (Jain et al. 2018). The pairwise ANI values are shown in Table 2. For yeasts, ANI values above 95% indicate that the two genomes may belong to the same species (Goris et al. 2007). In the case of the three *Blastobotrys* species, the ANI values seem consistent with the species classification, and only the two assemblies for *B. illinoisensis* present ANI > 95% (Table 2). Thus, we can conclude that the three *Blastobotrys* species are closely related but indeed different species based on whole-genome comparisons.

**Table 2:** Species genome sequence identity matrix. Average pairwise nucleotide identity (ANI, %) values between whole-genome assemblies of the three *Blastobotrys* species are shown

Species	*B. mokoenaii*	*B. illinoisensis* (PacBio)	*B. illinoisensis* (Illumina)	*B. malaysiensis*
*B. mokoenaii*	100	82.3	82.3	80.1
*B. illinoisensis* (PacBio)	82.3	100	99.9	80.4
*B. illinoisensis* (Illumina)	82.3	99.9	100	80.3
*B. malaysiensis*	80.1	80.4	80.4	100

### Gene annotation and CAZyme prediction

A total of 6069 (*B. mokoenaii*), 6177 (*B. illinoisensis* (PacBio)), and 7209 (*B. malaysiensis*) protein-coding genes were identified (Table 3). The vast majority of the genes (98–99%) could be clustered in orthogroups and were shared among the three species. Functional annotation of the predicted genes was performed with Interproscan, which detected a large fraction of genes (94–96%) that could be associated with functional information from one of the InterPro databases (Table 3). The annotation results reflect an overall gene number consistent with other yeast genomes (4700–6500; Shen et al. 2018). *B. malaysiensis* is perhaps an exception with a significantly higher gene number. This may be partly explained by the fragmented assembly, which could have caused genes to be split across multiple contigs (Denton et al. 2014). In fact, BUSCO fragmentation is highest in *B. malaysiensis* compared to the other species (4% vs 2.5–2.9%; Table 1), and mean gene length is also somewhat shorter in *B. malaysiensis* compared to the other species (Table 3). However, considering the larger genome size of *B. malaysiensis* (Table 1), it is also possible that the higher gene count in this species reflects genuine gene gains within this lineage.

**Table 3:** Functional annotation statistics for *Blastobotrys* species

Number/species	*B. mokoenaii*	*B. illinoisensis* (PacBio)	*B. malaysiensis*
Genes	6069	6177	7209
Mean gene length (bp)	1444	1465	1326
Genes w. functional annotation (%)	5828 (96)	5890 (95.4)	6780 (94)
Genes in orthogroups (%)	6010 (99)	6071 (98.3)	7041 (97.7)

The *Blastobotrys* genomes were also analyzed for predicted CAZymes involved in biomass decomposition using dbCAN3 (Zheng et al. 2023). All three *Blastobotrys* yeast genomes contained highly similar GH abundance, with a total CAZyme number of 213, 204, and 221 for *B. mokoenaii*, *B. illinoisensis*, and *B. malaysiensis*, respectively. All predicted CAZymes for each yeast are available at https://figshare.com/s/3b0f9f7bf805136ff794. For xylan degradation, all three yeasts contained a wide spectrum of predicted xylanolytic enzymes, including the GH11 xylanase which was previously characterized from *B. mokoenaii* (Ravn et al. 2023) and a subfamily GH30_7 enzyme with putative glucuronoxylanase activity (Table 4).

**Table 4:** Predicted CAZymes in *Blastobotrys* yeasts with activity for xylans. Xylanases are marked in bold

Polymer	*B. mokoenaii*	*B. illinoisensis*	*B. malaysiensis*
Xylans	CE1, CE4, GH3(8), GH5*, GH5_5, GH5_9(2), GH5_12, GH5_22(2), GH5_49, **GH11**, GH30_5, **GH30_7**, GH43_6, GH43_24, GH51_1, GH51_2(2), GH62, GH67, GH115	CE1, CE4, CE9, GH3(8), GH5_5(3), GH5_9(2), GH5_12(2), GH5_22, GH5_49, **GH11**, GH30_5, **GH30_7,** GH43_6, GH43_11 GH43_24, GH51_1, GH51_2(2), GH67, GH115	CE1, CE4, CE9, GH3(7), GH5_5(2), GH5_9(2), GH5_12(2), GH5_22, GH5_49, **GH11**, GH30_5, **GH30_7**, GH43_6, GH43_24, GH51_1, GH51_2(2), GH67, GH115
**Total CAZymes**	**213**	**204**	**221**

*GH5 enzymes have potential activity on many β-linked polysaccharides such as xylan, mannan, xyloglucan, and cellulose. Glycosyltransferases are not included (except in total CAZymes). *CAZymes*, Carbohydrate-Active EnZymes. *CE*, carbohydrate esterases; *GH*, glycoside hydrolases. Parentheses indicate gene copy number

### Growth on different carbon sources

**Fig. 1:**
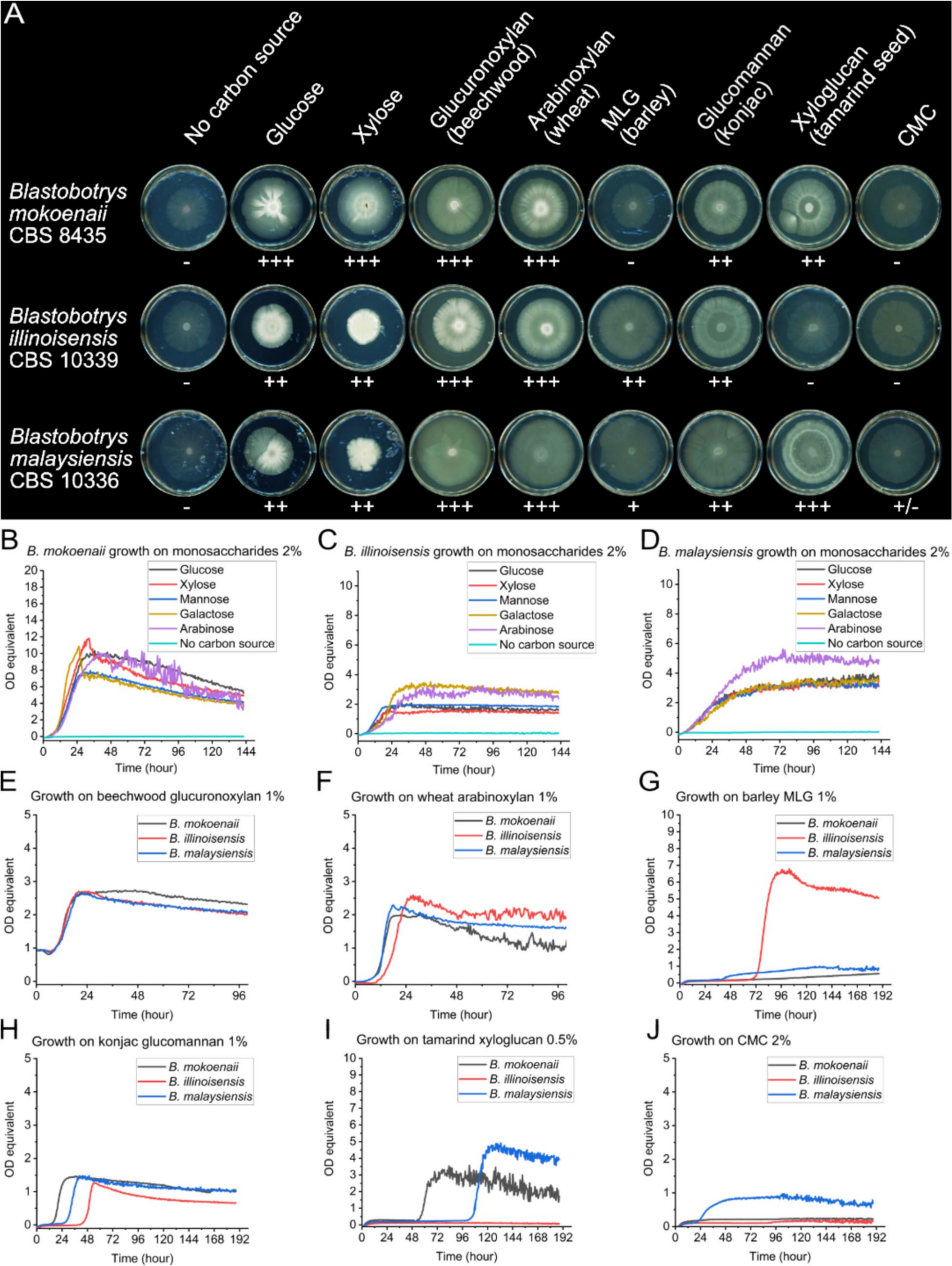
Growth on agar plates and in liquid cultures on different carbon sources. *Blastobotrys* growth on different monosaccharides or polysaccharides in agar plates (**A**) with 4 g L^−1^ carbon source after 5 weeks of growth at room temperature from a 20-µL culture drop with a starting OD = 5. Growth on plates was evaluated visually and categorized from − (no growth) to +  +  + (very good growth), depending on the diameter and density of the yeast colonies and hyphae morphologies. MLG = mixed-linkage glucan (barley), CMC = carboxymethyl cellulose. Growth of *B. mokoenaii* (**B**), *B. illinoisensis* (**C**), or *B. malaysiensis* (**D**) on different monosaccharides (20 g L^−1^). Growth of *Blastobotrys* yeast on beechwood glucuronoxylan 10 g L^−1^ (**E**), wheat arabinoxylan 10 g L^−1^ (**F**), konjac glucomannan 1 g L^−1^ (**G**), CMC 20 g L^−1^ (**H**), MLG 10 g L^−1^ (**I**), and tamarind xyloglucan 5 g L^−1^ (**J**). Variations in carbon source concentrations are due to high insolubility and viscosity at 10–20 g L^−1^ of some carbon sources. OD equivalent = optical density normalized from *S. cerevisiae* growth in Delft + 20 g L^−1^ glucose medium in a Growth-Profiler 960. All growth curves are means of biological triplicates

To connect genotypes and bioinformatic CAZyme predictions with growth phenotypes and enzyme activities, we evaluated the growth of the *Blastobotrys* yeasts on various carbon sources, including xylans. Growth was monitored on minimal medium agar plates (Fig. 1A) and in liquid cultures (Fig. 1B–J). All three yeasts showed robust growth on two types of xylans, beechwood GX and wheat arabinoxylan, and glucomannan, while exhibiting growth variations on β−1,3/1,4 mixed-linkage β-glucan (MLG) and xyloglucan. Thin hyphae were observed on both the plates lacking an added carbon source and those containing soluble carboxymethyl cellulose (CMC) (Fig. 1A), which was recorded as no growth. Additionally, see Supplementary Video S1 for documented growth over a 5-week timelapse.

**Fig. 2:**
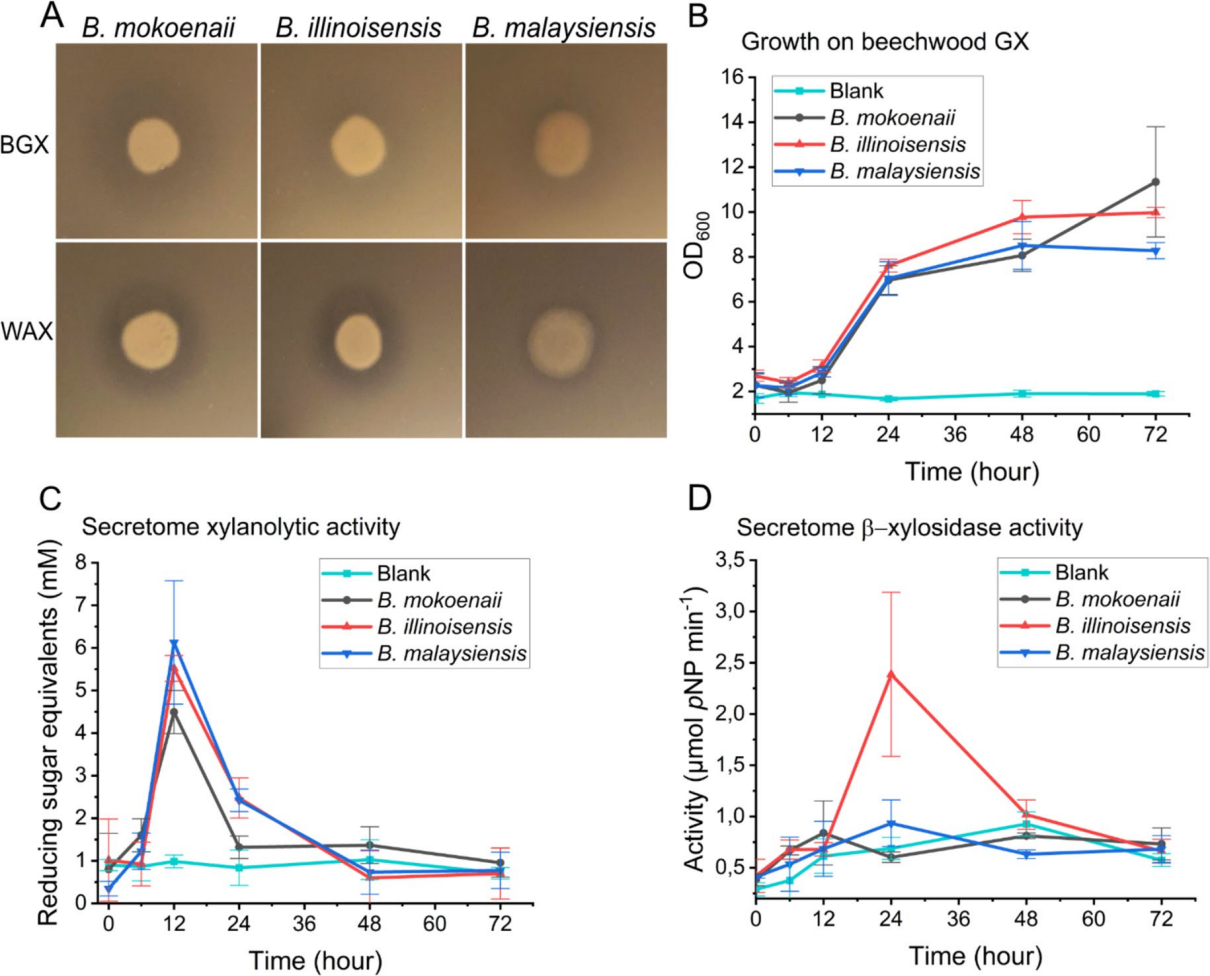
Xylanolytic activity of *Blastobotrys* yeasts. Halo formation in Delft medium + 4 g L^−1^ xylan in agar plates by *Blastobotrys* yeasts after 48 h of growth at room temperature (**A**). Growth of *Blastobotrys* yeasts in 30-mL Delft medium + 20 g L^−1^ beechwood GX in biological triplicates with error bars representing standard deviations (**B**). Beechwood GX hydrolysis activity, quantified by DNS assays, in *Blastobotrys* secretomes from yeasts grown in Delft + 20 g L^−1^ beechwood GX cultures in biological triplicates (**C**). β-Xylosidase activity, quantified using *p*NP-xylose, in *Blastobotrys* secretomes from yeasts grown in Delft medium + 20 g L^−1^ beechwood GX, in biological triplicates (**D**). BGX = beechwood glucuronoxylan, DNS = dinitro salicylate, GX = glucuronoxylan, WAX = wheat arabinoxylan

The liquid cultures largely confirmed the growth differences observed on agar plates. All three species exhibited robust growth on xylan, glucomannan, and the tested monosaccharides, although *B. illinoisensis* reached lower growth values on these simple sugars compared to the other two yeasts (Fig. 1B–G). *B. mokoenaii* and *B. malaysiensis* did not grow on CMC and MLG, whereas *B. illinoisensis* grew on MLG but showed limited growth on CMC and xyloglucan (Fig. 2I). The latter may in part be due to the absence of a GH12 xyloglucanase in *B. illinoisensis*, which is present in the other two species, along with differences in expression and regulation of other xyloglucan-related CAZymes. Overall, we can conclude that the *Blastobotrys* yeasts are markedly efficient at degrading and growing on xylan polymers.

### Enzymatic activity during growth in GX

The rapid growth of all three *Blastobotrys* yeasts on beechwood GX motivated us to characterize their enzymatic activities during growth on this carbon source. Clear halos formed around the growing yeast colonies for all three *Blastobotrys* species, indicating secretion of xylanolytic enzymes (Fig. 2A). In liquid cultures with beechwood GX, all three yeasts were able to significantly solubilize 20 g L^−1^ of the xylan after 72 h at 30 °C, and the strains grew to relatively high cell densities (OD_600_ = 8–11) (Fig. 2B). Secreted hydrolytic/xylanolytic activities peaked at approx. 12 h of incubation (Fig. 2C) for all three species, whereas only *B. illinoisensis* demonstrated pronounced extracellular β-xylosidase activity that appeared to peak around 24 h of incubation and then diminish (Fig. 2D).

### Secreted CAZymes during growth on glucuronoxylan

To identify which xylanolytic enzymes the three *Blastobotrys* yeasts secrete during growth in beechwood GX, we performed a proteomic analysis on secreted enzymes. The yeasts were grown in triplicates in minimal media containing 20 g L^−1^ beechwood GX as the sole carbon source and sampled after 72 h of incubation (Fig. 2B). The concentrated, cell-free samples were analyzed by LC–MS/MS and quantified with Minora semi-quantitative abundance analysis. Data are available via ProteomeXchange with identifier PXD061695.

In total, 213, 187, and 511 proteins were identified in the xylan-grown culture secretomes of *B. mokoenaii*, *B. illinoisensis*, and *B. malaysiensis*, respectively. Out of these, 105, 74, and 103 (49%, 40%, and 20%) had a predicted signal peptide indicating secretion, and 39, 23, and 36 (18%, 12%, and 7% of total proteins) were identified as CAZymes in the respective species. A full list of the CAZymes and the signal peptides is deposited at https://figshare.com/s/3b0f9f7bf805136ff794. The relatively large fractions of proteins not associated with signal peptides nor annotated as CAZymes suggest that some cells may have lysed during cultivation or sample preparation. Therefore, some caution is needed when interpreting the results, as not all the detected enzymes may have been secreted.

**Fig. 3:**
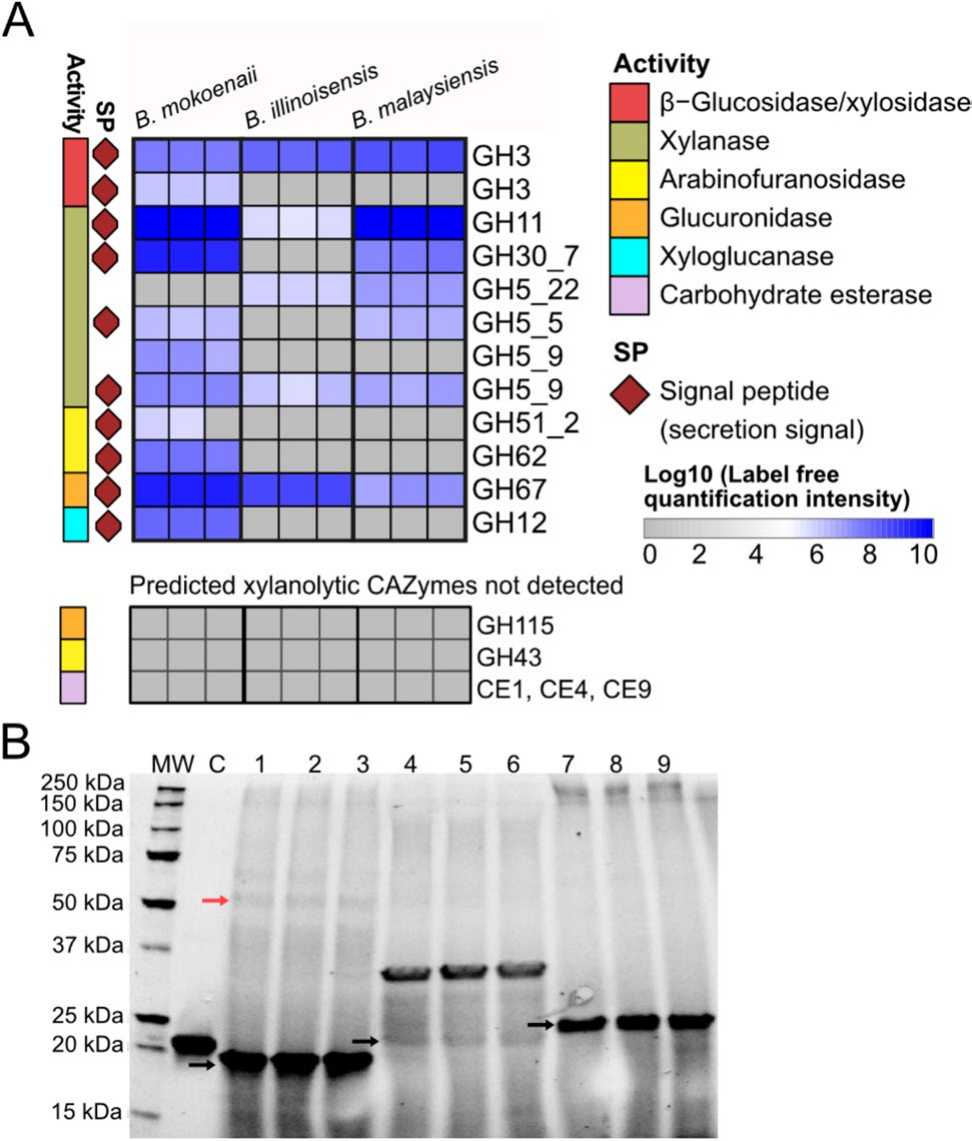
Secreted CAZymes during yeast growth on glucuronoxylan. The heatmap shows the detected abundances of annotated CAZyme proteins from three biological replicates of the secretomes from *B. mokoenaii*, *B. illinoisensis*, and *B. malaysiensis* during growth on GX. The colors in the heatmap indicate protein abundance, ranging from high (dark blue, log10 = 8–10 of label-free quantification) to low abundance (white, log10 = 4–5 of label-free quantification). Replicates with missing values are marked in gray (**A**). Sodium dodecyl-sulfate polyacrylamide gel electrophoresis (SDS-PAGE) analysis of proteins from 10 × concentrated secretomes from each biological replicate. MW = molecular protein weight ladder, C = control (recombinant *Bm*Xyn11A), lanes 1–3 = *B. mokoenaii*-concentrated secretomes, lanes 4–6 = *B. illinoisensis*-concentrated xylan secretomes, lanes 7–9 = *B. malaysiensis*-concentrated GX secretomes. Black arrows indicate the molecular weight predicted for GH11 proteins, and the red arrow indicates GH30_7 protein (**B**). The predicted molecular weights (without signal peptide sequence) of the GH11 xylanases are 21 kDa, 20 kDa, and 20 kDa for *B. mokoenaii, B. illinoisensis*, and *B. malaysiensis*, respectively, and 50 kDa GH30_7 for B. *mokoenaii*. CAZyme = Carbohydrate-Active EnZyme, GX = glucuronoxylan

Among the detected CAZymes, several enzymes with predicted activity on xylan were identified, most of which had predicted signal peptides (Fig. 3A). All three yeasts produced significant amounts of xylan main-chain degrading GH11 xylanases, and *B. mokoenaii* and *B. malaysiensis* also produced GH30_7 glucuronoxylanases*.* Additionally, the yeasts produced various enzymes from GH5, a polyspecific enzyme family associated with various activities including xylanase and β-xylosidase (Aspeborg et al. 2012; Huy et al. 2015). Notably, *B. mokoenaii* displayed a broader range of these putative xylan main-chain degrading proteins compared to the two other yeasts (Fig. 3A).

The high abundance of putative GH11 xylanases (~ 20 kDa) in the secretomes of *B. mokoenaii* and *B. malaysiensis* was also evident from SDS-PAGE analysis of 10 × concentrated secretomes (average protein concentration of 5–7 mg/mL protein) from xylan-enriched cultures (Fig. 3B). Additionally, zymograms performed on twin SDS-PAGE gels soaked in xylan and stained with Congo red revealed clear xylanolytic activity from re-folded GH11 enzymes (Supplementary Fig. S1B). A stained ~ 50-kDa protein band was also detected for *B. mokoenaii*, and in-gel proteomics confirmed this to be a GH30_7 xylanase (Supplementary Table S1). *B. illinoisensis* exhibited a fainter putative GH11 band compared to the other yeasts, and the protein had a slightly higher molecular weight than predicted, likely indicating protein glycosylation (Fig. 3B). Interestingly, the gel revealed another dominant protein band at ~ 35 kDa for *B. illinoisensis.* This band was absent in the secretomes from cultures grown in Delft medium supplemented with 20 g L^−1^ glucose or xylose (Supplementary Fig. S1D), suggesting it may be a protein associated with xylan metabolism. However, in-gel proteomics did not yield a definitive identification, assigning the protein either as a glycosylated GH16_18 glucanase (64% sequence similarity to an ortholog from *Sugiyamaella lignohabitans*, involved in cross-linking chitin to β-(1,6)-glucan in the yeast cell wall) or as an extracellular β-glycosidase (50% sequence similarity to an ortholog from *Yarrowia lipolytica*) (Supplementary Table S2). Moreover, it did not exhibit xylanolytic activity in the zymogram assay (Supplementary Fig. S1). Thus, a specific link to xylan metabolism for this enzyme remains unclear, and it may instead be involved in yeast cell wall re-modification.

The *Blastobotrys* yeasts also produced enzymes that putatively cleave off different types of sidechains from the xylan backbone. All three *Blastobotrys* species secreted a predicted GH67 α-glucuronidase to remove glucuronic acid residues from the extreme non-reducing end of xylooligosaccharides (Nurizzo et al. 2002). All three yeasts also possess a predicted GH115 α-glucuronidase and carbohydrate esterases (CEs), though none of these was detected in the secretomes. This indicates that α-glucuronidases and CEs may be cell-bound, which correlates well with the results in our previous study on *B. mokoenaii* (Ravn et al. 2023). *B. mokoenaii* also produced two arabinofuranosidases (GH51_2 and GH62) and an endo-β−1,4-glucanase (GH12) that are all typically associated with xyloglucan. Finally, we detected secreted GH3 β-xylosidases for all three species.

Along with xylanolytic enzymes, we also identified enzymes with predicted activities against fungal cell walls (β-glucans, chitin, lignin, starch, pectin, and proteoglycan), indicating growth-coupled remodeling of the yeast cell walls and hydrolysis of other polysaccharides. In particular, CAZymes that were secreted in high abundance include a putative GH65 trehalase and a GH16 extracellular glucosidase or β-(1,3)-glucanase, likely involved in chitin degradation or β-glucan fungal cell wall modification (Mouyna et al. 2016). An overview of all detected CAZymes with high abundance can be viewed in Supplementary Fig. S2.

Overall, the secretome results correlate well with the predominantly extracellular endo-xylanase activities and almost exclusively cell-bound β-xylosidase, α-arabinofuranosidase, α-glucuronidase, and esterase activities that we found for *B. mokoenaii* in our previous study (Ravn et al. 2023).

### *Bm*Xyn30A characterization and additive effects with *Bm*Xyn11A 

Considering the high abundance of GH11 enzymes in the secretomes of *B. mokoenaii* and *B. malaysiensis*, these xylanases are likely the primary contributors to the xylanolytic activity observed in these yeasts. We previously recombinantly expressed and characterized *Bm*Xyn11A from *B. mokoenaii* (Ravn et al. 2023), which shares 87–90% protein sequence similarity with the GH11 xylanases from *B. illinoisensis* and *B. malaysiensis* (Supplementary Fig. S3). As evident from the secretome analysis, the *Blastobotrys* yeasts also produced GH30_7 glucuronoxylanases (77–86% sequence identity). While GH11 xylanases are known to require three consecutive unsubstituted xylose units to cleave the xylan backbone, GH30_7 glucuronoxylanases require 4-*O*-methyl glucuronic acid substituents to bind and cleave the xylan backbone (Nakamichi et al. 2020). Thus, there may be additive or synergistic actions between the two xylanases, as well as with the other xylanolytic enzymes expressed. To investigate the activity of GH30_7 from *B. mokoenaii*, hereafter referred to as *Bm*Xyn30A, we recombinantly produced the protein in* P. pastoris* and characterized the purified enzyme’s activity, alone and in combination with other CAZymes (Supplementary Fig. S1H-I).

**Fig. 4:**
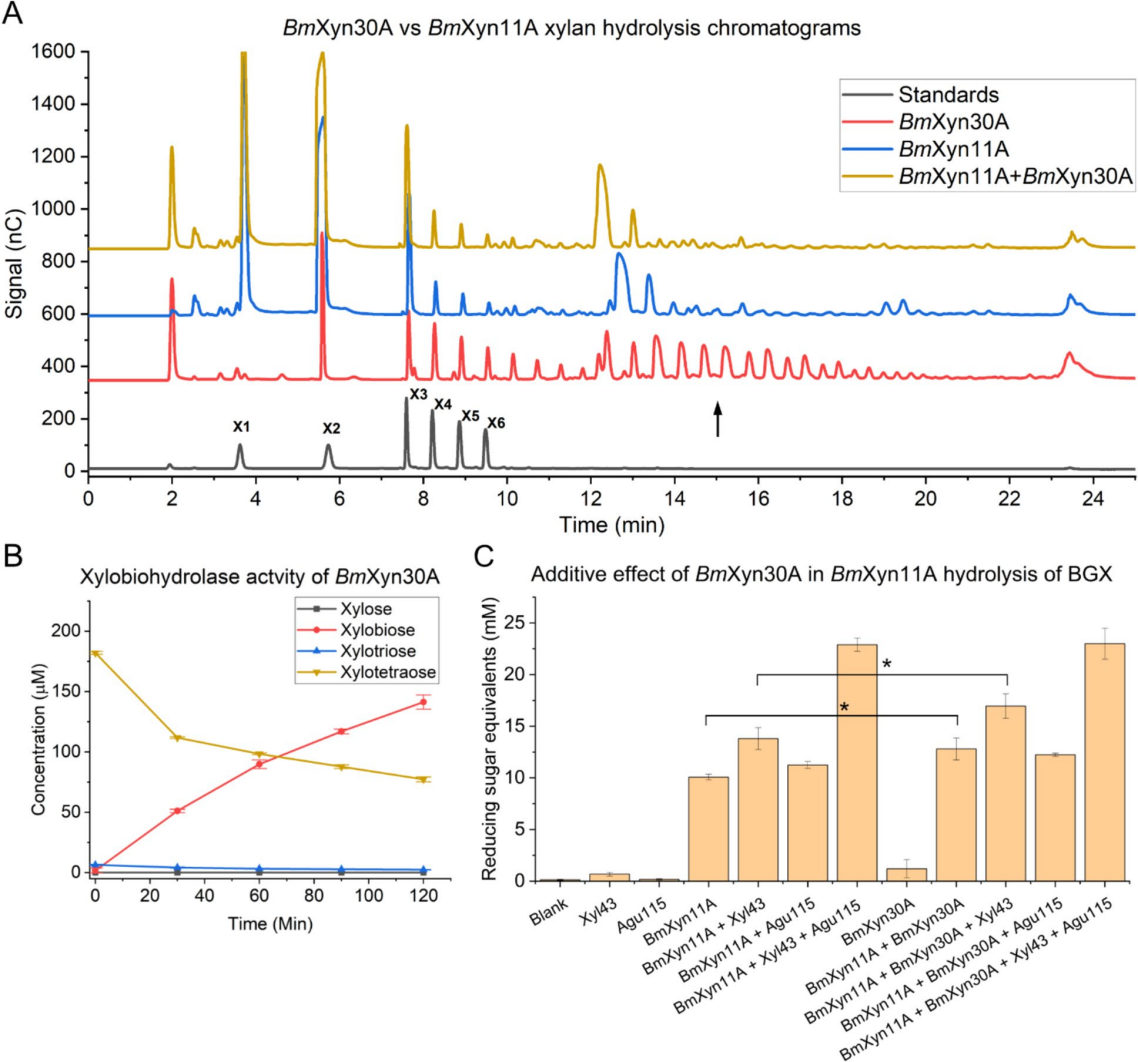
*Bm*Xyn30A xylan degradation assay and xylooligosaccharide analysis. Chromatogram comparing xylooligosaccharide profiles from beechwood GX hydrolysis by *Bm*Xyn30A showing more intense peaks at later retention time (indicated by black arrow), compared to *Bm*Xyn11A and the two enzymes combined (**A**). Xylobiohydrolase activity of *Bm*Xyn30A using 200-µL xylotetraose analyzed by HPAEC-PAD (**B**). Additive effects to *Bm*Xyn11A BGX hydrolysis with *Bm*Xyn30A after 16 h incubation in 10 g L^−1^ beechwood GX in 100 mM sodium acetate buffer pH 5 at 40 °C, 600 rpm, using 0.1 µM enzyme concentrations and DNS reducing sugar assays. Values are means of triplicates with standard deviations as error bars. Asterisks indicate statistical significance hydrolysis levels with *p*-values of 0.05 (*) considered significant (*n* = 3) and evaluated using one-way analysis of variance (ANOVA) with Tukey’s test (**C**). BGX = beechwood glucuronoxylan. GX = glucuronoxylan. HPAEC-PAD = high-performance anion-exchange chromatography coupled with pulsed amperometric detection

To compare the enzymatic activities of *Bm*Xyn30A with *Bm*Xyn11, xylooligosaccharide (XO) profiles were assessed after incubation in 20 g L^−1^ beechwood GX. *Bm*Xyn30A generated XO peaks with later retention times compared to *Bm*Xyn11, indicating production of glucuronic acid-branched XOs (Sanz Rodríguez et al. 2022) and/or XOs with larger degrees of polymerization than those produced by *Bm*Xyn11A (Fig. 4A). In addition, *Bm*Xyn30A acted as a xylobiohydrolase, specifically converting xylotetraose to xylobiose with a specific activity of 5.08 ± 0.17 µmol min^−1^ mg^−1^ for xylotetraose (Fig. 4B, Supplementary Fig. S4), highlighting the broad specificity and importance of this enzyme for glucuronoxylan metabolism in these strains.

To understand the interaction between these two types of xylanases, we combined *Bm*Xyn11A with *Bm*Xyn30A in 0.1 µM concentrations under optimal enzyme conditions (Supplementary figure S1I-H). As beechwood GX is substituted with glucuronic acid at an approximate ratio of 1:10 (Ebringerová and Heinze 2000), this relatively sparse substitution pattern may reflect the comparatively low amount of hydrolyzed reduced ends observed for *Bm*Xyn30A alone (Fig. 4C). Although modest, the additive effect of combining *Bm*Xyn30A with *Bm*Xyn11A in beechwood GX hydrolysis was significant, as evidenced by the increased production of reducing sugars (Fig. 4C). The combined hydrolysis yield (12.8 mM) was similar to the sum of individual enzymes (11.3 mM). Addition of commercial enzymes mimicking the activities of CAZymes found in the *B. mokoenaii* secretome, specifically a α-glucuronidase (Agu115) and a β-xylosidase (Xyl43), increased the overall release of reducing sugars. Notably, supplementing the *Bm*Xyn30A + *Bm*Xyn11A combination with Xyl43 led to a greater increase in reducing sugars than the *Bm*Xyn11A + Xyl43 pairing alone (Fig. 4C). The combination of all four enzymes yielded the highest levels of reducing sugars, although the inclusion of *Bm*Xyn30A did not contribute to a significant improvement in the presence of the other enzymes (Fig. 4C).

## Discussion

The three *Blastobotrys* yeasts characterized in this study—*B. mokoenaii*, *B. illinoisensis*, and *B. malaysiensis*—demonstrate exceptional growth on xylan compared to most other ascomycetous yeasts (Ravn et al. 2021). We show here that GX growth is accompanied by the production of a range of different CAZymes, with the secretomes notably enriched in enzymes exhibiting xylanolytic activity. Moreover, these yeasts not only deconstruct xylan, but must also possess metabolic pathways and transporter systems that enable them to efficiently take up and metabolize the resulting mono- and disaccharides. With the genomes of the *Blastobotrys* yeasts now fully annotated, there is an opportunity to explore their molecular systems for various synthetic biology applications, particularly in the development of new cell factories capable of utilizing the sugars from lignocellulosic hydrolysates as carbon and energy sources (annotated genomes available from SciLifeLab Data Repository: 10.17044/scilifelab.28606814).

In this study, we focused on the enzymes secreted by the *Blastobotrys* yeasts during growth on beechwood GX. The extracellular secretion of hydrolytic enzyme cocktails, or “secretomes,” is a well-established microbial strategy for nutrient acquisition and cell wall remodeling (Girard et al. 2013). This capability has also been extensively exploited in industrial applications, particularly with filamentous fungi such as *Aspergillus niger* and *Trichoderma reesei* (Bischof et al. 2016; Florencio et al. 2016). Understanding the composition and function of these enzyme systems, as well as their interactions with plant polysaccharides, holds great promise for advancing biotechnological applications in a bioeconomy framework (Filiatrault-Chastel et al. 2021). However, while analyzing the secretomes provides valuable insights, it overlooks the role of cell-bound enzymes in xylan degradation. In fact, previous studies on *B. mokoenaii* have demonstrated that this yeast preferentially expresses cell-attached α-glucuronidases, α-arabinofuranosidases, and β-xylosidases (Ravn et al. 2023), which likely also play a significant role in xylan processing of this yeast. It is highly plausible that the combination of secreted and cell-attached enzymes offers a strategic advantage for microorganisms like *Blastobotrys*, where secreted enzymes enable the extracellular breakdown of large polysaccharides, while cell-attached enzymes sequester the partially degraded products near the cell surface, facilitating efficient uptake and catabolism. The latter likely include predicted xylanolytic CAZymes that did not show up in the secretome such as GH115 α-glucuronidases and de-acylating carbohydrate esterases CE1, CE2, and CE4.

The comparison of the secretomes from a single sampling point (72 h of culturing in beechwood GX) across the three *Blastobotrys* species revealed both similarities and distinct differences in their secreted enzyme profiles. In hindsight, a time-course proteomics approach where the dynamic abundances of xylanolytic enzymes were tracked throughout growth could have provided a more comprehensive view of how *Blastobotrys* yeasts strategize glucuronoxylan degradation. Nonetheless, although genetic tools for deleting genes encoding xylanolytic enzymes in *Blastobotrys* yeasts are not yet available to directly validate their roles in xylan degradation, our findings suggest that at least *B. mokoenaii* and *B. malaysiensis* primarily rely on a secreted GH11 xylanase for xylan depolymerization. This prominence highlights the potential of using these *Blastobotrys* yeasts as natural expression hosts for xylanase production. In addition, *Bm*Xyn11A exhibits no detectable cellulase activity (Ravn et al. 2023), a xylanase characteristic sought after by the paper industry. As such, yeast-derived xylanases could degrade hemicelluloses while preserving cellulose structures, enhancing their utility in applications requiring selective enzymatic activity.

One of the most notable observations in this work was the hydrolysis of beechwood GX facilitated by the GH30_7 glucuronoxylanase. These enzymes appear to play a dual role in xylan degradation, contributing both to cleavage of the GX backbone at 4-*O*-methyl glucuronic acid moieties (Puchart et al. 2021; Šuchová et al. 2021) and to xylobiohydrolase activity. This bifunctional activity has been shown previously for other GH30_7 enzymes found in the filamentous fungi *Talaromyces cellulolyticus* and *Acremonium alcalophilum* and the bacteria *Thermothelomyces thermophilus* and *Erwinia chrysanthemi* (Katsimpouras et al. 2019; Nakamichi et al. 2019, 2020; Pentari et al. 2023, 2024; Urbániková et al. 2011). The yeast *Sugiyamaella lignohabitans* also produces a GH30_7 glucuronoxylanase that has been characterized (Šuchová et al. 2020, Šuchová, Chyba, et al., 2022), which, to our knowledge, makes *Bm*Xyn30A the second GH30_7 enzyme described in yeasts so far. The ion chromatogram results indicate that one role of *Bm*Xyn30A is to generate longer and/or more branched XOs compared to *Bm*Xyn11A. These attributes may have important implications in designing specific and tailored XOs within application fields such as renewable energy, bioplastics, and health (Karlsson et al. 2018; Procópio et al. 2022; Macedo et al. 2023).

## Conclusions

The three xylanolytic *Blastobotrys* yeasts characterized in this work primarily utilize extracellular GH11 xylanases to deconstruct beechwood GX. Moreover, they seem to employ GH30_7 glucuronoxylanases, which produce GlcA-substituted XOs. These findings expand our understanding of the xylanolytic strategy of ascomycetous yeasts. Future exploration of these enzymatic systems, for example, using time-course proteomic analyses for a refined picture, could pave the way for synthetic biology engineering strategies aimed at developing innovative cell factories capable of efficiently utilizing renewable lignocellulosic biomass resources.

## Supplementary Information

Below is the link to the electronic supplementary material.

Supplementary file1 (DOCX 2733 KB)

## Data Availability

All data is provided within the article and supplementary material or can otherwise be provided by the corresponding author upon reasonable request.   Data publicly available in repositories include: The raw PacBio reads and polished assembly for *B. illinoisensis* were submitted to the European Nucleotide Archive under project PRJEB84056 and are available at: https://www.ebi.ac.uk/ena/browser/text-search?query = PRJEB84056%20 Files containing the gene annotations for the three *Blastobotrys* species are available online at the SciLifeLab Data Repository and can be accessed at https://doi.org/10.17044/scilifelab.28606814.v1 Proteomics raw data are available via ProteomeXchange with identifier PXD061695 (https://www.ebi.ac.uk/pride/). Secretomic analyses for each yeast on CAZymes, signal peptides and eggNOG mapper annotations are available from: https://figshare.com/s/3b0f9f7bf805136ff794.

## References

[CR1] Aspeborg H, Coutinho PM, Wang Y, Brumer H, Henrissat B (2012) Evolution, substrate specificity and subfamily classification of glycoside hydrolase family 5 (GH5). BMC Evol Biol 12. 10.1186/1471-2148-12-186 10.1186/1471-2148-12-186 PMC3526467 22992189

[CR2] Banner A, Toogood HS, Scrutton NS (2021) Consolidated bioprocessing: synthetic biology routes to fuels and fine chemicals. Microorganisms 9(5):1072. 10.3390/microorganisms9051079 34069865 10.3390/microorganisms9051079 PMC8157379

[CR3] Bischof RH, Ramoni J, Seiboth B (2016) Cellulases and beyond: the first 70 years of the enzyme producer Trichoderma reesei. Microb Cell Fact 15. 10.1186/s12934-016-0507-6 10.1186/s12934-016-0507-6 PMC4902900 27287427

[CR4] Boekhout T, Amend AS, El Baidouri F, Gabaldón T, Geml J, Mittelbach M, Robert V, Tan CS, Turchetti B, Vu D, Wang QM, Yurkov A (2022) Trends in yeast diversity discovery. Fungal Divers 114:491–537. 10.1007/s13225-021-00494-6

[CR5] Bourque G, Burns KH, Gehring M, Gorbunova V, Seluanov A, Hammell M, Imbeault M, Izsvák Z, Levin HL, Macfarlan TS, Mager DL, Feschotte C (2018) Ten things you should know about transposable elements 06 Biological Sciences 0604 Genetics. Genome Biol 19. 10.1186/s13059-018-1577-z 10.1186/s13059-018-1577-z PMC6240941 30454069

[CR6] Chan PP, Lin BY, Mak AJ, Lowe TM (2021) TRNAscan-SE 2.0: improved detection and functional classification of transfer RNA genes. Nucleic Acids Res 49:9077–9096. 10.1093/nar/gkab688 34417604 10.1093/nar/gkab688 PMC8450103

[CR7] Chin CS, Alexander DH, Marks P, Klammer AA, Drake J, Heiner C, Clum A, Copeland A, Huddleston J, Eichler EE, Turner SW, Korlach J (2013) Nonhybrid, finished microbial genome assemblies from long-read SMRT sequencing data. Nat Methods 10:563–569. 10.1038/nmeth.2474 23644548 10.1038/nmeth.2474

[CR8] Collins T, Gerday C, Feller G (2005) Xylanases, xylanase families and extremophilic xylanases. FEMS Microbiol Rev 29:3–23. 10.1016/j.femsre.2004.06.005 15652973 10.1016/j.femsre.2004.06.005

[CR9] Curry TM, Peña MJ, Urbanowicz BR (2023) An update on xylan structure, biosynthesis, and potential commercial applications. Cell Surf 9. 10.1016/j.tcsw.2023.100101 10.1016/j.tcsw.2023.100101 PMC9898438 36748082

[CR10] Denton JF, Lugo-Martinez J, Tucker AE, Schrider DR, Warren WC, Hahn MW (2014) Extensive error in the number of genes inferred from draft genome assemblies. PLoS Comput Biol 10. 10.1371/journal.pcbi.1003998 10.1371/journal.pcbi.1003998 PMC4256071 25474019

[CR11] Drula E, Garron ML, Dogan S, Lombard V, Henrissat B, Terrapon N (2022) The carbohydrate-active enzyme database: functions and literature. Nucleic Acids Res 50:571–577. 10.1093/nar/gkab1045 10.1093/nar/gkab1045 PMC8728194 34850161

[CR12] Du Preez J, De Goede E, Myburgh J (2009) Blastobotrys mokoenaii: a thermotolerant yeast that produces extracellular endo-β-xylanase. N Biotechnol 25:S52. 10.1016/j.nbt.2009.06.265

[CR13] Ebringerová A, Heinze T (2000) Xylan and xylan derivatives - biopolymers with valuable properties, 1: naturally occurring xylans structures, isolation procedures and properties. Macromol Rapid Commun 21:542–556. 10.1002/1521-3927(20000601)21:9%3c542::AID-MARC542%3e3.0.CO;2-7

[CR14] Emms DM, Kelly S (2019) OrthoFinder: phylogenetic orthology inference for comparative genomics. Genome Biol 20. 10.1186/s13059-019-1832-y 10.1186/s13059-019-1832-y PMC6857279 31727128

[CR15] Filiatrault-Chastel C, Heiss-Blanquet S, Margeot A, Berrin JG (2021) From fungal secretomes to enzymes cocktails: the path forward to bioeconomy. Biotechnol Adv 52. 10.1016/j.biotechadv.2021.107833 10.1016/j.biotechadv.2021.107833 34481893

[CR16] Florencio C, Cunha FM, Badino AC, Farinas CS, Ximenes E, Ladisch MR (2016) Secretome analysis of *Trichoderma reesei* and *Aspergillus niger* cultivated by submerged and sequential fermentation processes: enzyme production for sugarcane bagasse hydrolysis. Enzyme Microb Technol 90:53–60. 10.1016/j.enzmictec.2016.04.011 27241292 10.1016/j.enzmictec.2016.04.011

[CR17] Formenti G, Abueg L, Brajuka A, Brajuka N, Gallardo-Alba C, Giani A, Fedrigo O, Jarvis ED (2022) Gfastats: conversion, evaluation and manipulation of genome sequences using assembly graphs. Bioinformatics 38:4214–4216. 10.1093/bioinformatics/btac460 35799367 10.1093/bioinformatics/btac460 PMC9438950

[CR18] Gabriel L, Brůna T, Hoff KJ, Ebel M, Lomsadze A, Borodovsky M, Stanke M (2024) BRAKER3: fully automated genome annotation using RNA-seq and protein evidence with GeneMark-ETP, AUGUSTUS, and TSEBRA. Genome Res 34:769–777. 10.1101/gr.278090.123 38866550 10.1101/gr.278090.123 PMC11216308

[CR19] Girard V, Dieryckx C, Job C, Job D (2013) Secretomes: the fungal strike force. Proteomics 13:597–608. 10.1002/pmic.201200282 23349114 10.1002/pmic.201200282

[CR20] Goris J, Konstantinidis KT, Klappenbach JA, Coenye T, Vandamme P, Tiedje JM (2007) DNA-DNA hybridization values and their relationship to whole-genome sequence similarities. Int J Syst Evol Microbiol 57:81–91. 10.1099/ijs.0.64483-0 17220447 10.1099/ijs.0.64483-0

[CR21] Hendriks ATWM, van Lier JB, de Kreuk MK (2018) Growth media in anaerobic fermentative processes: the underestimated potential of thermophilic fermentation and anaerobic digestion. Biotechnol Adv 36:1–13. 10.1016/j.biotechadv.2017.08.004 28870855 10.1016/j.biotechadv.2017.08.004

[CR22] Huy ND, Le NC, Seo JW, Kim DH, Park SM (2015) Putative endoglucanase PcGH5 from Phanerochaete chrysosporium is a β-xylosidase that cleaves xylans in synergistic action with endo-xylanase. J Biosci Bioeng 119:416–420. 10.1016/j.jbiosc.2014.09.012 25300189 10.1016/j.jbiosc.2014.09.012

[CR23] Jain C, Rodriguez-R LM, Phillippy AM, Konstantinidis KT, Aluru S (2018) High throughput ANI analysis of 90K prokaryotic genomes reveals clear species boundaries. Nat Commun 9. 10.1038/s41467-018-07641-9 10.1038/s41467-018-07641-9 PMC6269478 30504855

[CR24] Jia L, Budinova GALG, Takasugi Y, Noda S, Tanaka T, Ichinose H, Goto M, Kamiya N (2016) Synergistic degradation of arabinoxylan by free and immobilized xylanases and arabinofuranosidase. Biochem Eng J 114:268–275. 10.1016/j.bej.2016.07.013

[CR25] Jones P, Binns D, Chang HY, Fraser M, Li W, McAnulla C, McWilliam H, Maslen J, Mitchell A, Nuka G, Pesseat S, Quinn AF, Sangrador-Vegas A, Scheremetjew M, Yong SY, Lopez R, Hunter S (2014) InterProScan 5: genome-scale protein function classification. Bioinformatics 30:1236–1240. 10.1093/bioinformatics/btu031 24451626 10.1093/bioinformatics/btu031 PMC3998142

[CR26] Karlsson EN, Schmitz E, Linares-Pastén JA, Adlercreutz P (2018) Endo-xylanases as tools for production of substituted xylooligosaccharides with prebiotic properties. Appl Microbiol Biotechnol 102:9081–9088. 10.1007/s00253-018-9343-4 30196329 10.1007/s00253-018-9343-4 PMC6208967

[CR27] Katsimpouras C, Dedes G, Thomaidis NS, Topakas E (2019) A novel fungal GH30 xylanase with xylobiohydrolase auxiliary activity. Biotechnol Biofuels 12. 10.1186/s13068-019-1455-2 10.1186/s13068-019-1455-2 PMC6511221 31110561

[CR28] Kojima K, Sunagawa N, Mikkelsen NE, Hansson H, Karkehabadi S, Samejima M, Sandgren M, Igarashi K (2022) Comparison of glycoside hydrolase family 3 β-xylosidases from basidiomycetes and ascomycetes reveals evolutionarily distinct xylan degradation systems. J Biol Chem 298. 10.1016/j.jbc.2022.101670 10.1016/j.jbc.2022.101670 PMC8913315 35120929

[CR29] Kriventseva EV, Kuznetsov D, Tegenfeldt F, Manni M, Dias R, Simão FA, Zdobnov EM (2019) OrthoDB v10: sampling the diversity of animal, plant, fungal, protist, bacterial and viral genomes for evolutionary and functional annotations of orthologs. Nucleic Acids Res 47:D807–D811. 10.1093/nar/gky1053 30395283 10.1093/nar/gky1053 PMC6323947

[CR30] Kuznetsov D, Tegenfeldt F, Manni M, Seppey M, Berkeley M, Kriventseva EV, Zdobnov EM (2023) OrthoDB v11: annotation of orthologs in the widest sampling of organismal diversity. Nucleic Acids Res 51:D445–D451. 10.1093/nar/gkac998 36350662 10.1093/nar/gkac998 PMC9825584

[CR31] Macedo JVC, Abe MM, Sanvezzo PB, Grillo R, Branciforti MC, Brienzo M (2023) Xylan-starch-based bioplastic formulation and xylan influence on the physicochemical and biodegradability properties. Polym Bull 80:8067–8092. 10.1007/s00289-022-04385-x

[CR32] Magrane M, Consortium UP (2011) UniProt Knowledgebase: a hub of integrated protein data. Database 2011. 10.1093/database/bar009 10.1093/database/bar009 PMC3070428 21447597

[CR33] Manni M, Berkeley MR, Seppey M, Simão FA, Zdobnov EM (2021) BUSCO update: novel and streamlined workflows along with broader and deeper phylogenetic coverage for scoring of eukaryotic, prokaryotic, and viral genomes. Mol Biol Evol 38:4647–4654. 10.1093/molbev/msab199 34320186 10.1093/molbev/msab199 PMC8476166

[CR34] McCleary BV, McGeough P (2015) A Comparison of polysaccharide substrates and reducing sugar methods for the measurement of endo-1,4-β-xylanase. Appl Biochem Biotechnol 177:1152–1163. 10.1007/s12010-015-1803-z 26289020 10.1007/s12010-015-1803-z PMC4633439

[CR35] Mendonça M, Barroca M, Collins T (2023) Endo-1,4-β-xylanase-containing glycoside hydrolase families: characteristics, singularities and similarities. Biotechnol Adv 65. 10.1016/j.biotechadv.2023.108148 10.1016/j.biotechadv.2023.108148 37030552

[CR36] Mnich E, Bjarnholt N, Eudes A, Harholt J, Holland C, Jørgensen B, Larsen FH, Liu M, Manat R, Meyer AS, Mikkelsen JD, Motawia MS, Muschiol J, Møller BL, Møller SR, Perzon A, Petersen BL, Ravn JL, Ulvskov P (2020) Phenolic cross-links: building and de-constructing the plant cell wall. Nat Prod Rep 37:919–961. 10.1039/c9np00028c 31971193 10.1039/c9np00028c

[CR37] Mouyna I, Aimanianda V, Hartl L, Prevost MC, Sismeiro O, Dillies MA, Jagla B, Legendre R, Coppee JY, Latgé JP (2016) GH16 and GH81 family β-(1,3)-glucanases in *Aspergillus fumigatus* are essential for conidial cell wall morphogenesis. Cell Microbiol 18:1285–1293. 10.1111/cmi.12630 27306610 10.1111/cmi.12630

[CR38] Naidu DS, Hlangothi SP, John MJ (2018) Bio-based products from xylan: a review. 179:28–41. 10.1016/j.carbpol.2017.09.064 10.1016/j.carbpol.2017.09.064 29111052

[CR39] Nakamichi Y, Fouquet T, Ito S, Watanabe M, Matsushika A, Inoue H (2019) Structural and functional characterization of a bifunctional GH30-7 xylanase B from the filamentous fungus *Talaromyces cellulolyticus*. J Biol Chem 294:4065–4078. 10.1074/jbc.RA118.007207 30655295 10.1074/jbc.RA118.007207 PMC6422087

[CR40] Nakamichi Y, Watanabe M, Matsushika A, Inoue H (2020) Substrate recognition by a bifunctional GH30-7 xylanase B from *Talaromyces cellulolyticus*. FEBS Open Bio 10:1180–1189. 10.1002/2211-5463.12873 32359208 10.1002/2211-5463.12873 PMC7262913

[CR41] Nurizzo D, Nagy T, Gilbert HJ, Davies GJ (2002) The structural basis for catalysis and specificity of the *Pseudomonas cellulosa*-glucuronidase, GlcA67A. Structure 10:547–556. 10.1016/S0969-2126(02)00742-6 11937059 10.1016/s0969-2126(02)00742-6

[CR42] Opulente DA, LaBella AL, Harrison MC, Wolters JF, Liu C, Li Y, Kominek J, Steenwyk JL, Stoneman HR, VanDenAvond J, Miller CR, Langdon QK, Silva M, Gonçalves C, Ubbelohde EJ, Li Y, Buh KV, Jarzyna M, Haase MAB, Rosa CA, ČCadež N, Libkind D, DeVirgilio JH, Hulfachor AB, Kurtzman CP, Sampaio JP, Gonçalves P, Zhou X, Shen XX, Groenewald M, Rokas A, Hittinger CT (2004) Genomic factors shape carbon and nitrogen metabolic niche breadth across Saccharomycotina yeasts. Science 384(6694):eadj4503. 10.1126/science.adj4503 10.1126/science.adj4503 PMC11298794 38662846

[CR43] Paysan-Lafosse T, Blum M, Chuguransky S, Grego T, Pinto BL, Salazar GA, Bileschi ML, Bork P, Bridge A, Colwell L, Gough J, Haft DH, Letunić I, Marchler-Bauer A, Mi H, Natale DA, Orengo CA, Pandurangan AP, Rivoire C, Sigrist CJA, Sillitoe I, Thanki N, Thomas PD, Tosatto SCE, Wu CH, Bateman A (2023) InterPro in 2022. Nucleic Acids Res 51:D418–D427. 10.1093/nar/gkac993 36350672 10.1093/nar/gkac993 PMC9825450

[CR44] Pentari C, Kosinas C, Nikolaivits E, Dimarogona M, Topakas E (2024) Structural and molecular insights into a bifunctional glycoside hydrolase 30 xylanase specific to glucuronoxylan. Biotechnol Bioeng. 10.1002/bit.28731 38678481 10.1002/bit.28731

[CR45] Pentari C, Zerva A, Dimarogona M, Topakas E (2023) The xylobiohydrolase activity of a GH30 xylanase on natively acetylated xylan may hold the key for the degradation of recalcitrant xylan. Carbohydr Polym 305. 10.1016/j.carbpol.2022.120527 10.1016/j.carbpol.2022.120527 36737185

[CR46] Procópio DP, Kendrick E, Goldbeck R, Damasio AR de L, Franco TT, Leak DJ, Jin YS, Basso TO (2022) Xylo-oligosaccharide utilization by engineered *Saccharomyces cerevisiae* to produce ethanol. Front Bioeng Biotechnol 10. 10.3389/fbioe.2022.825981 10.3389/fbioe.2022.825981 PMC8886126 35242749

[CR47] Puchart V, Biely P (2022) Microbial xylanolytic carbohydrate esterases. Essays Biochem EBC2022012:83–97. 10.1007/1-4020-5377-0_6 10.1042/EBC20220129 36468678

[CR48] Puchart V, Šuchová K, Biely P (2021) Xylanases of glycoside hydrolase family 30 – an overview. Biotechnol Adv 47. 10.1016/j.biotechadv.2021.107704 10.1016/j.biotechadv.2021.107704 33548454

[CR49] Ravn JL, Engqvist MKM, Larsbrink J, Geijer C (2021) CAZyme prediction in ascomycetous yeast genomes guides discovery of novel xylanolytic species with diverse capacities for hemicellulose hydrolysis. Biotechnol Biofuels 14. 10.1186/s13068-021-01995-x 10.1186/s13068-021-01995-x PMC8254220 34215291

[CR50] Ravn JL, Ristinmaa AS, Coleman T, Larsbrink J, Geijer C (2023) Yeasts have evolved divergent enzyme strategies to deconstruct and metabolize xylan. Microbiol Spectr 11. 10.1128/spectrum.00245-23 10.1128/spectrum.00245-23 PMC10269524 37098941

[CR51] Rohman A, Dijkstra BW, Puspaningsih NNT (2019) β-xylosidases: structural diversity, catalytic mechanism, and inhibition by monosaccharides. Int J Mol Sci 20. 10.3390/ijms20225524 10.3390/ijms20225524 PMC6887791 31698702

[CR52] Ruben D, Sanya A, Onésime D, Passoth V, Maiti MK, Chattopadhyay A, Khot MB (2021) Yeasts of the *Blastobotrys* genus are promising platform for lipid-based fuels and oleochemicals production. Appl Microbiol Biotechnol 2021:4879–4897. 10.1007/s00253-021-11354-3 10.1007/s00253-021-11354-3 34110474

[CR53] Sanz Rodríguez E, Díaz-Arenas GL, Makart S, Ghosh D, Patti AF, Garnier G, Tanner J, Paull B (2022) Determination of xylooligosaccharides produced from enzymatic hydrolysis of beechwood xylan using high-performance anion-exchange chromatography tandem mass spectrometry. J Chromatogr A 1666. 10.1016/j.chroma.2022.462836 10.1016/j.chroma.2022.462836 35108629

[CR54] Shen XX, Opulente DA, Kominek J, Zhou X, Steenwyk JL, Buh KV, Haase MAB, Wisecaver JH, Wang M, Doering DT, Boudouris JT, Schneider RM, Langdon QK, Ohkuma M, Endoh R, Takashima M, Manabe R, Čadež N, Libkind D, Rosa CA, DeVirgilio J, Hulfachor AB, Groenewald M, Kurtzman CP, Hittinger CT, Rokas A (2018) Tempo and mode of genome evolution in the budding yeast subphylum. Cell 175:1533-1545.e20. 10.1016/j.cell.2018.10.023 30415838 10.1016/j.cell.2018.10.023 PMC6291210

[CR55] Simmons TJ, Mortimer JC, Bernardinelli OD, Pöppler AC, Brown SP, DeAzevedo ER, Dupree R, Dupree P (2016) Folding of xylan onto cellulose fibrils in plant cell walls revealed by solid-state NMR. Nat Commun 7. 10.1038/ncomms13902 10.1038/ncomms13902 PMC5187587 28000667

[CR56] Sjöström E (1993) Wood Polysaccharides. Wood Chemistry 51–70. 10.1016/b978-0-08-092589-9.50007-3

[CR57] Šuchová K, Puchart V, Spodsberg N, Mørkeberg Krogh KBR, Biely P (2020) A novel GH30 xylobiohydrolase from *Acremonium alcalophilum* releasing xylobiose from the non-reducing end. Enzyme Microb Technol 134. 10.1016/j.enzmictec.2019.109484 10.1016/j.enzmictec.2019.109484 32044031

[CR58] Šuchová K, Puchart V, Spodsberg N, Mørkeberg Krogh KBR, Biely P (2021) Catalytic diversity of GH30 xylanases. Molecules 26. 10.3390/molecules26154528 10.3390/molecules26154528 PMC8347883 34361682

[CR59] Šuchová K, Chyba A, Hegyi Z, Rebroš M, Puchart V (2022a) Yeast GH30 xylanase from *Sugiyamaella lignohabitans* is a glucuronoxylanase with auxiliary xylobiohydrolase activity. Molecules 27. 10.3390/molecules27030751 10.3390/molecules27030751 PMC8840591 35164030

[CR60] Šuchová K, Fehér C, Ravn JL, Bedő S, Biely P, Geijer C (2022b) Cellulose- and xylan-degrading yeasts: enzymes, applications and biotechnological potential. Biotechnol Adv 59. 10.1016/j.biotechadv.2022.107981 10.1016/j.biotechadv.2022.107981 35580749

[CR61] Tõlgo M, Hüttner S, Rugbjerg P, Thuy NT, Thanh VN, Larsbrink J, Olsson L (2021) Genomic and transcriptomic analysis of the thermophilic lignocellulose-degrading fungus *Thielavia terrestris* LPH172. Biotechnol Biofuels 14. 10.1186/s13068-021-01975-1 10.1186/s13068-021-01975-1 PMC8176577 34082802

[CR62] Urbániková Ä, Vršanská M, Mãrkeberg Krogh KBR, Hoff T, Biely P (2011) Structural basis for substrate recognition by *Erwinia chrysanthemi* GH30 glucuronoxylanase. FEBS J 278:2105–2116. 10.1111/j.1742-4658.2011.08127.x 21501386 10.1111/j.1742-4658.2011.08127.x

[CR63] Visagie CM, Boekhout T, Theelen B, Dijksterhuis J, Yilmaz N, Seifert KA (2023) Da Vinci’s yeast: *Blastobotrys davincii* f.a., sp. nov. Yeast 40:7–31. 10.1002/yea.3816 36168284 10.1002/yea.3816 PMC10108157

[CR64] Vu D, Groenewald M, Szöke S, Cardinali G, Eberhardt U, Stielow B, de Vries M, Verkleij GJM, Crous PW, Boekhout T, Robert V (2016) DNA barcoding analysis of more than 9 000 yeast isolates contributes to quantitative thresholds for yeast species and genera delimitation. Stud Mycol 85:91–105. 10.1016/j.simyco.2016.11.007 28050055 10.1016/j.simyco.2016.11.007 PMC5192050

[CR65] Wiśniewski JR, Zougman A, Nagaraj N, Mann M (2009) Universal sample preparation method for proteome analysis. Nat Methods. 6(5):359–362. 10.1038/nmeth.1322 19377485 10.1038/nmeth.1322

[CR66] Zexer N, Paradiso A, Nong D, Haviland ZK, Hancock WO, Anderson CT (2024) Xylan inhibition of cellulase binding and processivity observed at single-molecule resolution. RSC Sustainability 2:1118–1127. 10.1039/d4su00006d

[CR67] Zheng J, Ge Q, Yan Y, Zhang X, Huang L, Yin Y (2023) DbCAN3: automated carbohydrate-active enzyme and substrate annotation. Nucleic Acids Res 51:115–121. 10.1093/nar/gkad328 10.1093/nar/gkad328 PMC10320055 37125649

