# Supplementary material for: Genomic and secretomic analyses of Blastobotrys yeasts reveal key xylanases for biomass decomposition
Source: Appl Microbiol Biotechnol. 2025 Aug 1;109:175. doi: 10.1007/s00253-025-13556-5 (PMC12316802; doi:10.1007/s00253-025-13556-5)
Supplement: Supplementary file 1 [file 253_2025_13556_MOESM1_ESM.docx]

# **Supplementary material**

***Applied Microbiology and Biotechnology***

**Genomic and secretomic analyses of *Blastobotrys* yeasts reveal key xylanases for biomass decomposition**

Jonas Ravn^1,3*^, Amanda S. Ristinmaa^1^, Scott Mazurkewich^1,2^, Guilherme B. Dias^4^, Johan Larsbrink^1,2^, Cecilia Geijer^1*^.

^1^Department of Life Sciences, Chalmers University of Technology, 412 96, Gothenburg, Sweden.

^2^Wallenberg Wood Science Center, Teknikringen 56-58, 100 44, Stockholm, Sweden.

^3^RISE Research Institutes of Sweden, Division of Bioeconomy, Sweden, Department of Food Research & Innovation, Frans Perssons väg 6, SE-412 76, Gothenburg, Sweden.

^4^Department of Cell and Molecular Biology, National Bioinformatics Infrastructure Sweden, Science for Life Laboratory, Uppsala University, Husargatan 3, 75237 Uppsala, Sweden.

*Corresponding authors: Jonas Ravn [jonas.ravn@ri.se](mailto:jonas.ravn@ri.se), Cecilia Geijer [cecilia.geijer@chalmers.se](mailto:cecilia.geijer@chalmers.se)

**Supplementary Figure S1.** SDS-PAGE with concentrated secretomes from BGX cultures with lane 1-3= *B. mokoenaii* concentrated secretomes, lane 4-6= *B. illinoisensis* concentrated xylan secretomes, lane 7-9= *B. malaysiensis* concentrated xylan secretomes (A). Zymogram analysis of xylan-soaked and subsequently Congo Red-stained gel (with same lane samples as in A), with the red arrow indicating GH30_7 enzyme from *Blastobotrys mokoenaii* (B). Endo H-treated *B. illinoisensis* 35 kDa protein (C). Glucose (lane 1-3) and xylose (lane 4-6) cultures in minimal media with *B. illinoisensis* (D). Protein band gel cut outs for in-gel proteomics indicated by blue arrows and boxes with 1-2= *B. mokoenaii* concentrated secretomes, lane 3-4= *B. illinoisensis* concentrated xylan secretomes, lane 5-6= *B. malaysiensis* concentrated xylan secretomes (E). SDS-PAGE analysis of purified recombinantly produced *Bm*Xyn30A at different dilutions with lane 1=10-fold, lane 2= 20-fold, lane 3= 50-fold and lane 4= 100-fold protein dilutions (F). Purified *Bm*Xyn11A with lane 1= 10-fold and lane 2=100-fold protein dilution (G). Effect of pH (H) and temperature (I) on BGX xylanase activity of *Bm*Xyn30A in triplicates and means are plotted with their standard deviations. BGX= beechwood glucuronoxylan.

**Supplementary Figure S2.** The heatmap shows abundance of the annotated CAZyme proteins secreted from three biological replicates of *B. mokoenaii* xylan secretome, *B. illinoisensis* xylan secretome and *B. malaysiensis* xylan secretome. The colors in the heatmap indicate protein abundance, ranging from high (dark blue, log10= 8-10 of LFQ) to low abundance (white, log10= 4-5 of LFQ). Replicates with missing values are marked in grey. CAZyme= carbohydrate active enzyme, LFQ= label free quantification. SP= signal peptide (secretion signal detected).

**Supplementary Figure S3.** Protein sequence alignments of predicted GH11 and GH30_7 from *Blastobotrys* yeasts using CLUSTAL multiple sequence alignment by MUSCLE. Bi= *Blastobotrys illinoisensis,* Bm= *Blastobotrys mokoenaii,* Bma= *Blastobotrys malaysiensis.*

**Supplementary Figure S4: Xylotetraose hydrolysis by *Bm*Xyn30A.** Progress curve of assays of 0.3 nmol of *Bm*Xyn30A with 200 μM xylotetraose (X4, p) in 50 mM sodium acetate at pH 5, detected by high-performance anion-exchange chromatography coupled with pulsed amperometric detection. Hydrolysis of xylotetraose leads to production of xylobiose (X2, l) with xylotriose (X2, n) concentrations remaining unchanged and no detectable xylose being produced. Assays were performed in triplicate and means are plotted with their standard deviations. The lines are linear regressions taken during steady state between 30-120 mins. Note that the initial rate between 0-30 min is faster than that during the steady state and thus the specific activity reported is a lower approximation.

**Supplementary Video S1** Timelapse over 5 weeks of yeast growth on different carbon sources.

**Supplementary List S1**

**Protein sequence of *Bm*Xyn30A**

>*Bm*Xyn30A

MLSQIIAAALTFSTISLAAHSSATVTVDLNIQYQSVDGFGCSQAFQRAEEIFGKDGLSPKNQSHLLDLLFSQELGAGFTILRNGIGSSNSSDKNFMNSIEPFSPGSPSNPPHYTWDNYDSGQLQLAKEAKKRGLATLYADAWSAPGYMKSNNDENWGGYLCGVSGTDCESGDWMQAYADYLVQYWKYYRNAGVPPTHLGFINEPEEVVSYASMETNGTQAAEFIKVLGQTLEREGIDIELTCCDSNGWQHQENLMAGLQAVGPDGKSGEDYLSVITGHGYASPPNFPLSTKLRTWQTEWTDLSGHYTPYTFYSTGAAGEGLTWAENIQTAFVNANVSAFLGWIGAENATVNSGLITLLGDEIVPSKRFWAMASFSKFVRPGAVRVESTSSDPSLKVSAFQNKDGIIALQVINNSTSSAAMSIDFGVPVRGNHQWKYVVPWITNNDHDLDAQKSIPIGKDGKFEAVVPSKSLVSFVSSHK

**Codon optimized DNA sequence of *Bm*Xyn30A**

>*Bm*GH30_7_pPICZalphaA

AGATCTAACATCCAAAGACGAAAGGTTGAATGAAACCTTTTTGCCATCCGACATCCACAGGTCCATTCTCACACATAAGTGCCAAACGCAACAGGAGGGGATACACTAGCAGCAGACCGTTGCAAACGCAGGACCTCCACTCCTCTTCTCCTCAACACCCACTTTTGCCATCGAAAAACCAGCCCAGTTATTGGGCTTGATTGGAGCTCGCTCATTCCAATTCCTTCTATTAGGCTACTAACACCATGACTTTATTAGCCTGTCTATCCTGGCCCCCCTGGCGAGGTTCATGTTTGTTTATTTCCGAATGCAACAAGCTCCGCATTACACCCGAACATCACTCCAGATGAGGGCTTTCTGAGTGTGGGGTCAAATAGTTTCATGTTCCCCAAATGGCCCAAAACTGACAGTTTAAACGCTGTCTTGGAACCTAATATGACAAAAGCGTGATCTCATCCAAGATGAACTAAGTTTGGTTCGTTGAAATGCTAACGGCCAGTTGGTCAAAAAGAAACTTCCAAAAGTCGGCATACCGTTTGTCTTGTTTGGTATTGATTGACGAATGCTCAAAAATAATCTCATTAATGCTTAGCGCAGTCTCTCTATCGCTTCTGAACCCCGGTGCACCTGTGCCGAAACGCAAATGGGGAAACACCCGCTTTTTGGATGATTATGCATTGTCTCCACATTGTATGCTTCCAAGATTCTGGTGGGAATACTGCTGATAGCCTAACGTTCATGATCAAAATTTAACTGTTCTAACCCCTACTTGACAGCAATATATAAACAGAAGGAAGCTGCCCTGTCTTAAACCTTTTTTTTTATCATCATTATTAGCTTACTTTCATAATTGCGACTGGTTCCAATTGACAAGCTTTTGATTTTAACGACTTTTAACGACAACTTGAGAAGATCAAAAAACAACTAATTATTCGAAACGATGAGATTTCCTTCAATTTTTACTGCTGTTTTATTCGCAGCATCCTCCGCATTAGCTGCTCCAGTCAACACTACAACAGAAGATGAAACGGCACAAATTCCGGCTGAAGCTGTCATCGGTTACTCAGATTTAGAAGGGGATTTCGATGTTGCTGTTTTGCCATTTTCCAACAGCACAAATAACGGGTTATTGTTTATAAATACTACTATTGCCAGCATTGCTGCTAAAGAAGAAGGGGTATCTCTCGAGAAAAGAGAGGCTGAAGCTGAATTCGCCCACTCGAGCGCGACCGTAACAGTTGACTTAAACATCCAGTATCAATCTGTAGATGGTTTTGGATGCAGCCAAGCATTCCAGCGGGCTGAAGAAATTTTTGGCAAAGACGGCCTCTCACCGAAGAACCAGTCTCATTTACTAGATCTTTTGTTCTCCCAAGAGCTAGGCGCAGGATTTACTATTTTACGAAACGGGATCGGGAGTTCCAATTCGTCGGACAAAAATTTTATGAACTCTATAGAACCATTTTCCCCGGGCTCTCCAAGCAACCCCCCACACTACACTTGGGACAATTATGATTCTGGACAACTACAGCTCGCCAAAGAGGCTAAGAAGCGTGGACTCGCCACTTTATACGCTGACGCTTGGTCGGCCCCTGGATATATGAAGAGCAATAATGATGAAAACTGGGGCGGTTACCTTTGTGGTGTGAGTGGCACAGACTGCGAATCTGGTGACTGGATGCAGGCATACGCGGATTACTTGGTGCAATATTGGAAATACTATCGGAATGCCGGGGTGCCTCCGACGCACCTAGGTTTTATAAATGAGCCTGAAGAGGTAGTTTCGTACGCATCGATGGAAACTAATGGAACCCAAGCTGCGGAGTTTATTAAAGTTCTGGGTCAGACACTGGAGCGAGAAGGGATCGATATCGAGCTGACCTGCTGTGACTCCAACGGGTGGCAGCACCAAGAGAACCTTATGGCGGGGCTCCAGGCGGTGGGACCGGATGGGAAATCCGGTGAAGATTATCTGTCAGTCATAACAGGTCATGGTTATGCGTCACCCCCAAATTTTCCCTTGTCCACGAAGTTGCGTACTTGGCAAACCGAATGGACCGATCTAAGTGGCCATTATACTCCTTACACGTTCTATTCTACGGGTGCAGCGGGAGAAGGACTTACTTGGGCCGAAAACATACAGACGGCATTCGTCAATGCCAATGTAAGTGCGTTCTTGGGGTGGATCGGTGCGGAGAACGCCACAGTAAACAGTGGCTTGATCACGCTACTCGGCGATGAGATTGTTCCCAGCAAGAGGTTTTGGGCCATGGCTTCATTCTCAAAATTCGTCAGACCGGGCGCAGTACGCGTAGAGTCTACTTCATCCGATCCCAGCCTGAAGGTCAGTGCATTCCAGAACAAGGACGGGATTATAGCTTTACAAGTGATTAATAACAGTACAAGCTCTGCAGCTATGTCAATCGACTTCGGAGTCCCTGTTAGAGGCAATCATCAATGGAAATACGTCGTTCCGTGGATAACCAACAACGATCACGACCTTGACGCCCAGAAGTCAATACCAATTGGGAAAGACGGAAAATTCGAAGCTGTGGTTCCCAGCAAGTCCCTTGTGAGTTTTGTCTCGAGCCATAAGGTCGACCATCATCATCATCATCATTGAGTTTGTAGCCTTAGACATGACTGTTCCTCAGTTCAAGTTGGGCACTTACGAGAAGACCGGTCTTGCTAGATTCTAATCAAGAGGATGTCAGAATGCCATTTGCCTGAGAGATGCAGGCTTCATTTTTGATACTTTTTTATTTGTAACCTATATAGTATAGGATTTTTTTTGTCATTTTGTTTCTTCTCGTACGAGCTTGCTCCTGATCAGCCTATCTCGCAGCTGATGAATATCTTGTGGTAGGGGTTTGGGAAAATCATTCGAGTTTGATGTTTTTCTTGGTATTTCCCACTCCTCTTCAGAGTACAGAAGATTAAGTGAGACCTTCGTTTGTGCGGATCCCCCACACACCATAGCTTCAAAATGTTTCTACTCCTTTTTTACTCTTCCAGATTTTCTCGGACTCCGCGCATCGCCGTACCACTTCAAAACACCCAAGCACAGCATACTAAATTTCCCCTCTTTCTTCCTCTAGGGTGTCGTTAATTACCCGTACTAAAGGTTTGGAAAAGAAAAAAGAGACCGCCTCGTTTCTTTTTCTTCGTCGAAAAAGGCAATAAAAATTTTTATCACGTTTCTTTTTCTTGAAAATTTTTTTTTTTGATTTTTTTCTCTTTCGATGACCTCCCATTGATATTTAAGTTAATAAACGGTCTTCAATTTCTCAAGTTTCAGTTTCATTTTTCTTGTTCTATTACAACTTTTTTTACTTCTTGCTCATTAGAAAGAAAGCATAGCAATCTAATCTAAGGGCGGTGTTGACAATTAATCATCGGCATAGTATATCGGCATAGTATAATACGACAAGGTGAGGAACTAAACCATGGCCAAGTTGACCAGTGCCGTTCCGGTGCTCACCGCGCGCGACGTCGCCGGAGCGGTCGAGTTCTGGACCGACCGGCTCGGGTTCTCCCGGGACTTCGTGGAGGACGACTTCGCCGGTGTGGTCCGGGACGACGTGACCCTGTTCATCAGCGCGGTCCAGGACCAGGTGGTGCCGGACAACACCCTGGCCTGGGTGTGGGTGCGCGGCCTGGACGAGCTGTACGCCGAGTGGTCGGAGGTCGTGTCCACGAACTTCCGGGACGCCTCCGGGCCGGCCATGACCGAGATCGGCGAGCAGCCGTGGGGGCGGGAGTTCGCCCTGCGCGACCCGGCCGGCAACTGCGTGCACTTCGTGGCCGAGGAGCAGGACTGACACGTCCGACGCGGCCCGACGGGTCCGAGGCCTCGGAGATCCGTCCCCCTTTTCCTTTGTCGATATCATGTAATTAGTTATGTCACGCTTACATTCACGCCCTCCCCCCACATCCGCTCTAACCGAAAAGGAAGGAGTTAGACAACCTGAAGTCTAGGTCCCTATTTATTTTTTTATAGTTATGTTAGTATTAAGAACGTTATTTATATTTCAAATTTTTCTTTTTTTTCTGTACAGACGCGTGTACGCATGTAACATTATACTGAAAACCTTGCTTGAGAAGGTTTTGGGACGCTCGAAGGCTTTAATTTGCAAGCTGGAGACCAACATGTGAGCAAAAGGCCAGCAAAAGGCCAGGAACCGTAAAAAGGCCGCGTTGCTGGCGTTTTTCCATAGGCTCCGCCCCCCTGACGAGCATCACAAAAATCGACGCTCAAGTCAGAGGTGGCGAAACCCGACAGGACTATAAAGATACCAGGCGTTTCCCCCTGGAAGCTCCCTCGTGCGCTCTCCTGTTCCGACCCTGCCGCTTACCGGATACCTGTCCGCCTTTCTCCCTTCGGGAAGCGTGGCGCTTTCTCATAGCTCACGCTGTAGGTATCTCAGTTCGGTGTAGGTCGTTCGCTCCAAGCTGGGCTGTGTGCACGAACCCCCCGTTCAGCCCGACCGCTGCGCCTTATCCGGTAACTATCGTCTTGAGTCCAACCCGGTAAGACACGACTTATCGCCACTGGCAGCAGCCACTGGTAACAGGATTAGCAGAGCGAGGTATGTAGGCGGTGCTACAGAGTTCTTGAAGTGGTGGCCTAACTACGGCTACACTAGAAGAACAGTATTTGGTATCTGCGCTCTGCTGAAGCCAGTTACCTTCGGAAAAAGAGTTGGTAGCTCTTGATCCGGCAAACAAACCACCGCTGGTAGCGGTGGTTTTTTTGTTTGCAAGCAGCAGATTACGCGCAGAAAAAAAGGATCTCAAGAAGATCCTTTGATCTTTTCTACGGGGTCTGACGCTCAGTGGAACGAAAACTCACGTTAAGGGATTTTGGTCATGAGATC

**Proteomics of *Bm*Xyn30A and 35 kDa from *B. illinoisensis*.**

**Supplementary Table S1:**

In-gel proteomics fragments table for gel fragment containing GH30_7 protein from *B. mokoenaii*

| **Accession gene** | **Coverage [%]** | **# peptides** | **# PSMs** | **# unique Peptides** | **MW [kDa]** | **dbCAN** |
| --- | --- | --- | --- | --- | --- | --- |
| gene=snap_masked-NODE_14_length_262008_cov_38.1084_ID_27-processed-gene-1.140 CDS=1-888 | 36 | 8 | 20 | 8 | 28.6 |  |
| gene=snap_masked-NODE_4_length_585455_cov_36.5712_ID_7-processed-gene-4.197 CDS=1-1131 | 22 | 6 | 12 | 6 | 42.2 |  |
| gene=snap_masked-NODE_1_length_970412_cov_36.0026_ID_1-processed-gene-1.127 CDS=1-1143 | 36 | 8 | 12 | 8 | 42.4 |  |
| gene=genemark-NODE_31_length_153071_cov_36.322_ID_61-processed-gene-0.204 CDS=1-1152 | 17 | 6 | 11 | 6 | 42* | **GH30_7(1-380)** |

*Note the small discrepancy of molecular weight (MW) since dbCAN3 did not predict full protein due to a methionine in protein sequence.

**Supplementary Table S2:**

In-gel proteomics fragments table for ~35 kDa protein in *B. illinoisensis*

| **Accession gene** | **Coverage [%]** | **# Peptides** | **# PSMs** | **# Unique Peptides** | **MW [kDa]** | **dbCAN** |
| --- | --- | --- | --- | --- | --- | --- |
| g1741.t1 | 52 | 15 | 176 | 15 | 40 |  |
| g955.t2 | 15 | 4 | 15 | 4 | 28,8 |  |
| g2400.t1 | 13 | 5 | 15 | 5 | 48,7 | GH16_18 |
| g3054.t1 | 1 | 1 | 8 | 1 | 208,2 |  |
